# CFD, energy, and exergy analysis and sustainability indicators of tilapia fish strips drying using an evacuated tubes indirect solar dryer

**DOI:** 10.1038/s41598-025-11230-4

**Published:** 2025-07-17

**Authors:** Amena Ali Alsakran, Omar Shahat Younis, András Székács, Omar Saeed, Mohamed Hamdy Eid, Ali Majrashi, Atef Fathy Ahmed, Aml Abubakr Tantawy, Abdallah Elshawadfy Elwakeel

**Affiliations:** 1 Department of Biology, College of Science and Humanities in Al-Kharj, Prince Sattam Bin Abdulaziz University, 11942 Al-Kharj, Saudi Arabia; 2 Food Manufacturing Engineering and Packaging Department, Agriculture Research Center, Food Technology Research Institute, Giza, Egypt; 3 Agro-Environmental Research Centre, Institute of Environmental Sciences, Hungarian University of Agriculture and Life Sciences, Páter Károly u. 1, Gödöllő, 2100 Hungary; 4 Doctoral School of Environmental Science, Hungarian University of Agriculture and Life Sciences (MATE), Páter Károly u. 1, Gödöllő, 2100 Hungary; 5 Institute of Environmental Management, Faculty of Earth Science, University of Miskolc, Miskolc-Egyetemváros, 3515 Hungary; 6 Geology Department, Faculty of Science, Beni-Suef University, Beni-Suef, 65211 Egypt; 7 Department of Biology, College of Science, Taif University, P.O. Box 11099, 21944 Taif, Saudi Arabia; 8 Food Science Department, Faculty of Agriculture, Beni-Suef University, Beni-Suef, 65211 Egypt; 9 Agricultural Engineering Department, Faculty of Agriculture and Natural Resources, Aswan University, Aswan, 81528 Egypt

**Keywords:** Computational fluid dynamics (CFD), Energy and exergy analysis, energy and exergy efficiency, Improvement potential (IP), Waste exergy ratio (WER), Sustainability index (SI), Solar dryer, Solar thermal energy, Environmental impact

## Abstract

This study evaluates the performance of an evacuated tube indirect solar dryer (ETISD) for drying tilapia strips at three thicknesses (4, 8, and 12 mm) using computational fluid dynamics (CFD), energy-exergy analysis, and sustainability indicators. CFD simulations were employed to analyze airflow patterns, temperature distribution, and velocity profiles inside the drying room (DR) across five air velocities (0.02–0.06 m/s). The optimal air flow rate of 0.03 m^3^/s provided a uniform drying temperature of 74.82 °C, at solar noon. Simulations over two consecutive drying days (8 a.m.–5 p.m.) further assessed thermal and aerodynamic behavior, enhancing system optimization. Energy analysis revealed that the evacuated tube solar collector (ETSC) achieved a maximum input energy of 1311.8 W and useful energy of 682.5 W, with energy efficiencies of 44.5–51.2% (ETSC) and 16.18–21.57% (ETISD). Exergy efficiencies ranged from 8.51 to 21.99% (ETSC) and 29.23–84.76% (ETISD), highlighting thermodynamic performance. Sustainability indicators, including improvement potential (IP) (2.71–6.69 W), waste exergy ratio (WER) (1.15–1.36), and sustainability index (SI) (1.09–1.28), demonstrated the system’s environmental and operational viability. These findings underscore the ETISD’s effectiveness for sustainable tilapia drying, balancing energy efficiency, thermal performance, and ecological impact.

## Introduction

Drying is a crucial post-harvest process that extends the shelf life of agricultural products by reducing moisture content, thereby preventing spoilage and microbial growth^1–3^. Traditional drying methods, such as open-air sun drying or electric dehydrators, often face challenges like contamination, uneven drying, and high energy consumption^4–6^. In contrast, solar drying presents an efficient and sustainable alternative by utilizing renewable solar energy to dehydrate crops while maintaining nutritional quality^7,8^. SDs significantly reduce dependence on conventional electricity and fossil fuels, lowering both operational costs and carbon emissions^9–11^. They are particularly beneficial in rural and off-grid areas, where access to reliable power is limited. By optimizing airflow and temperature control, SDs ensure faster, more uniform drying compared to open-air methods, improving product quality and marketability^12–14^.

SDs can be classified based on their design, airflow mechanism, and energy utilization. The main categories include direct, indirect, mixed-mode, and hybrid SDs. Direct SDs expose the fish directly to sunlight within an enclosed chamber, allowing natural heat and airflow to remove moisture. Indirect SDs use solar collectors to heat air separately before passing it over the fish, preventing direct UV exposure and preserving product color and nutrients. Mixed-mode SDs combine both direct and indirect heating for faster drying rates. Hybrid SDs integrate supplementary energy sources (e.g., biomass or electric heaters) to ensure continuous operation during cloudy weather^10,15,16^. Further classifications include passive SDs (relying on natural convection) and active SDs (using fans for forced airflow). Each type has advantages depending on climate, scale of production, and desired product quality. By selecting the appropriate SDs, processors can optimize efficiency, reduce losses, and produce higher-quality dried fish for commercial markets^7,17,18^.

CFD analysis plays a pivotal role in optimizing the design and performance of SDs by simulating airflow, temperature distribution, and heat transfer within the drying system. Unlike traditional trial-and-error methods, CFD provides a cost-effective and time-efficient way to visualize and analyze complex thermal and fluid dynamics processes, enabling precise modifications for enhanced efficiency^19,20^. CFD helps identify hotspots, uneven drying zones, and airflow obstructions, allowing engineers to refine dryer geometry, vent placement, and insulation strategies. This leads to improved heat retention, uniform moisture removal, and reduced energy waste^21,22^. Additionally, CFD simulations can evaluate the impact of different operating conditions—such as varying solar radiation intensity (SRI), air velocity, and load capacity—without physical prototyping. By leveraging CFD, researchers can develop high-performance SDs tailored to specific crops and climatic conditions, maximizing drying rates while minimizing thermal losses^23,24^. Numerous studies were conducted to study the CFD of many types of SDs including, Sajadiye and Saberian^25^ used the CFD to study the effect of weather on temperature variation inside a SD in Ahvaz-Iran; Gonçalves et al.^26^ studied the CFD to analysis of the behavior of the air inside the device, and optimization of the sludge drying by calculating the thermal efficiency and drying efficiency of the equipment; Singh et al.^27^ the indirect type SD is studied and computational fluid dynamics is employed to simulate the process. Ansys fluent CFD is used to simulate and obtain dynamic and thermal performance of the dryer at different operating conditions (mass flow rate). The predicted results are validated with the help of experimental results; Roman-Roldan et al.^28^ used the CFD used the CFD for homogeneous solar drying of developed new air recirculation system; Suryavanshi1 and Ranade^29^ analyzed the performance of an evacuated tube solar collector of SD for drying agriculture products using CFD; Chavan et al.^30^, used the CFD analysis to optimize the sizing and location of the solar fan to make this grain dryer energy efficient; Thomas et al.^31^ studied the behavior of air in the collector for different inclinations angles, height of the drying chamber and chimney; and Güler et al.^32^; Getahun et al.^19^ reviewed recent advances in solar drying of fruits and vegetables, emphasizing that CFD had been widely used to study airflow, heat, and mass transfer for optimizing dryer design and operation. However, most CFD studies had not included product quality modeling, and the review suggested that future CFD-based evaluations should be able to predict both drying performance and product quality for more comprehensive optimization; Iranmanesh et al.^33^ investigated a solar cabinet dryer equipped with an evacuated tube solar collector and PCM thermal storage, using both CFD modeling and experiments at different air flow rates. The study found that using PCM increased the input thermal energy and achieved a maximum drying efficiency of 39.9% at 0.025 kg/s, with CFD and experimental results in good agreement. The use of PCM did not adversely affect the quality of the dried product; Madhankumar et al.^34^ reviewed the latest developments in solar dryer technologies, particularly indirect solar dryers with fins and thermal energy storage units. The review found that these systems, when analyzed with CFD, showed improved heat transfer and moisture removal kinetics, making them efficient and economically viable for food processing industries and farmers; Shekata et al.^35^ discussed recent advancements in indirect solar dryers and associated thermal energy storage. The review highlighted that CFD had been a valuable tool for optimizing dryer design and operation while maintaining product quality. It encouraged further research to enhance performance, energy efficiency, and scalability for sustainable agriculture; and Chouikhi et al.^36^ evaluated an indirect-mode forced convection solar dryer equipped with a PV/T air collector, using CFD modeling to simulate air temperature and velocity. The study found that CFD predictions closely matched experimental data, with average daily efficiencies of 30.9% for the collector, 15.2% for the dryer, and 8.7% for the PV panel, confirming the reliability of CFD for predicting system performance. On the other hand, energy analysis evaluates the thermal efficiency and heat utilization of SDs, while exergy analysis assesses the quality of energy conversion, identifying irreversible losses and inefficiencies in the system. Together, these analyses provide a comprehensive understanding of dryer performance, enabling improvements in design and operation^8,37,38^. Energy analysis helps quantify useful heat transfer and drying rates, ensuring optimal resource use. Meanwhile, exergy analysis pinpoints where energy degradation occurs—such as in collectors, airflow systems, or drying chambers—guiding technical enhancements to minimize waste^39–44^. This dual approach is critical for developing cost-effective, high-efficiency SDs that reduce energy consumption and operational costs. Furthermore, energy-exergy studies support sustainability by improving dryer designs for rural and off-grid applications, where energy access is limited^45,46^.

Numerous studies have evaluated the energy and exergetic performance of various SDs for agricultural product dehydration. Mugi and Chandramohan^47^ compared forced and natural convection in an indirect SD for okra drying, finding forced convection superior, with mean solar air collector (SAC) and drying efficiencies of 74.98% and 24.95%, respectively, versus 61.49% and 20.13% for natural convection. Exergy outflow was 1.04–46.85 W (forced) and 1.13–50.94 W (natural), while SAC exergy efficiencies averaged 2.03% (forced) and 2.44% (natural). The IP under forced convection ranged from 0.0095 to 10.51 W, with SI and WER values of 0.06–17.05. Selimefendigil et al.^48^ enhanced a greenhouse SD with nanoparticle-enhanced paraffin thermal storage, reporting exergy efficiencies of 3.45% (nanoparticle-aided) and 2.74% (baseline) at 0.016 kg/s airflow, and 3.01% versus 2.40% at 0.010 kg/s. Ekka et al.^49^ tested a mixed-mode SD with dual double-pass SACs on cluster figs, observing total exergy efficiencies of 18.8–41.4%, with higher airflow reducing exergy losses (SI: 1.26–1.71). Şevik et al.^38^ achieved energy efficiencies of 1.15–26.46% using a double-pass SD with optional infrared assistance. Chowdhury et al.^50^ analyzed a tunnel SD for jackfruit leather, noting SAC efficiencies of 27.45–42.50% and SD efficiencies of 32.34–65.30% (100–600 W/m^2^ irradiance). System-wide energy efficiency reached 42.47%, with exergetic efficiencies of 32–69% (SAC) and 41.42% (drying unit). Shringi et al.^51^ achieved 67.06–88.24% exergy efficiency in a PCM-equipped SD for garlic, while Panwar^52^ reported 55.35–79.35% for leaves in a natural convection SD. Ndukwu et al.^53^ used sodium sulfate decahydrate/NaCl storage, achieving 66.67–96.09% exergy efficiency. Kesavan et al.^54^ studied a triple-pass SD for potatoes (2.8–87.02% exergy efficiency), and Karthikeyan and Murugavelh^55^ reported 23.25–73.31% for turmeric in a mixed-mode tunnel SD. Tiwari and Tiwari^56^ evaluated a mixed-mode greenhouse SD (19.11–28.96% SAC exergy efficiency), and Abdelkader et al.^57^ achieved 8.1–11.9% in a carbon nanotube-enhanced SD.

Drying is a vital method for preserving tilapia fish, particularly in regions like Aswan, Egypt, where fish from Lake Nasser represents a crucial source of food and income. As one of the largest man-made lakes in the world, Lake Nasser supports a thriving fishery, with tilapia being the most commercially significant species due to its abundance, nutritional value, and consumer preference^43,44,58^. However, the high temperatures and remote fishing areas in Upper Egypt pose significant challenges to fish storage and transportation. Drying offers a cost-effective, energy-efficient solution for extending shelf life, minimizing post-harvest losses, and enabling broader market access both locally and nationally^59–61^. Moreover, improved drying technologies can enhance product quality, hygiene, and economic value, supporting food security and livelihoods in rural communities. As demand for dried fish grows in Egypt and neighboring regions, optimizing drying methods for tilapia from Lake Nasser becomes increasingly essential^62^. Thus, the aim of this study is to apply variable SRI throughout the process of tilapia fish strips for an Egyptian climate in a series of CFD models in order to simulate the temperature and air velocity inside the drying room of the developed ETISD throughout a drying period. Also, the effect of the air volume flow rate of the exhaust fan on air temperature and velocity inside the drying room was studied. Additionally, of energy and exergy for both the SD and DR, and an estimation of the sustainable indicators for the SD. The results of this study are also useful for observing the critical high temperatures and air velocity inside the drying room based on the air volume flow rate of the exhaust fan. Also, the time intervals of critical high temperatures that occur in Egypt can be enhanced by adjusting the air volume flow rate of the exhaust fan.

## Materials and methods

### Description of the developed ETISD

**Fig. 1:**
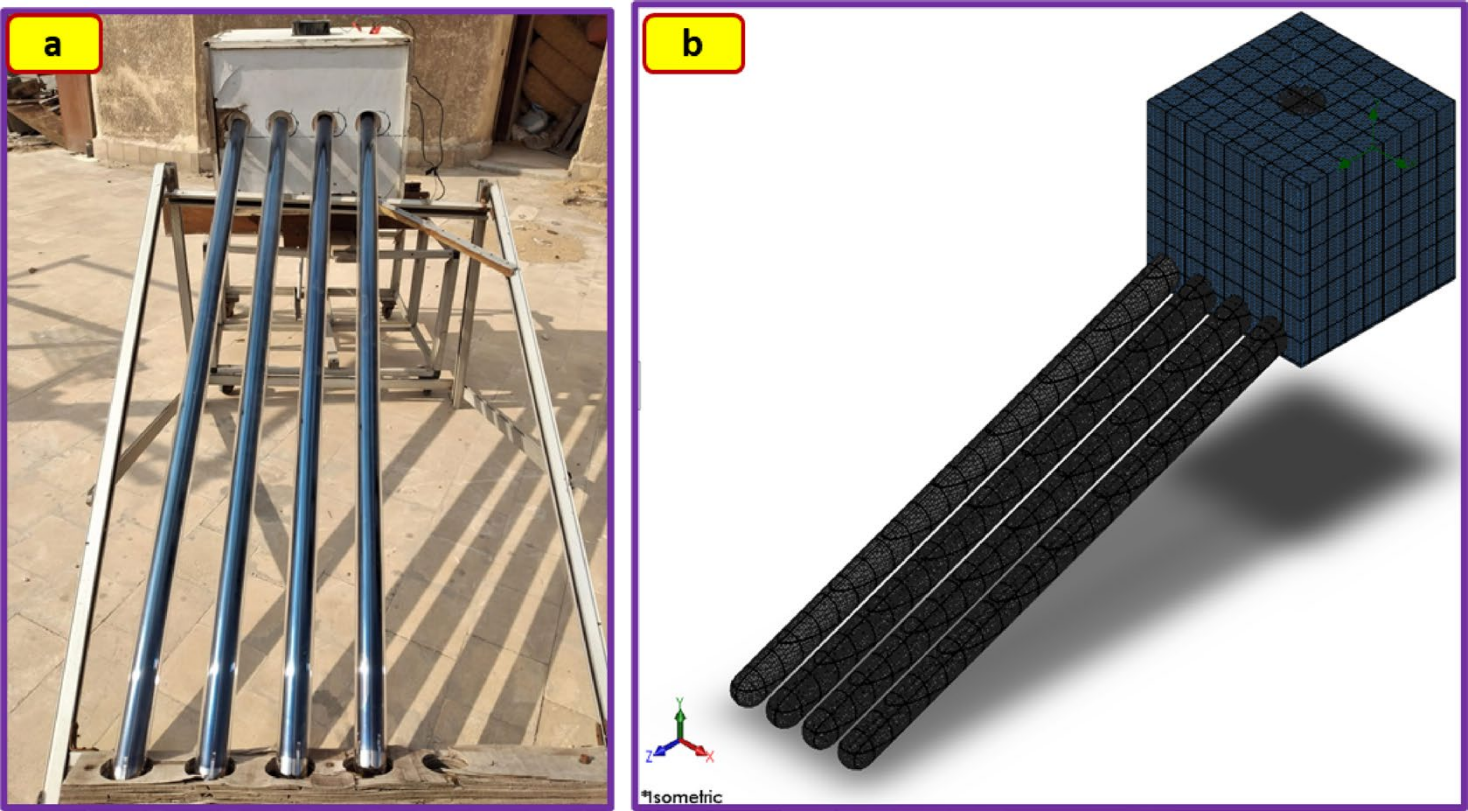
(**a**) Real and (**b**) meshing of the ETISD.

The constructed ETSC consists of four borosilicate glass vacuum tubes, that have a dimension of 58 mm in diameter, and 1800 mm in length (Fig. 1). The evacuated tubes were arranged within a wooden framework at a 30° angle, and at the upper sides, they were connected to a drying chamber of 600 mm × 600 mm × 600 mm. The upper copper end of the evacuated tubes is installed inside an internal manifold at the lower end of the drying chamber. Air enters the drying chamber from the side vent, passes through all the copper points, and then flows through the manifold. This method ensures the highest possible performance. The velocity of the air exhaust fan (model: Extech AN100, China) was controlled using a volt regulator unit to operate at a different velocity.

### Experiment setup

**Fig. 2:**
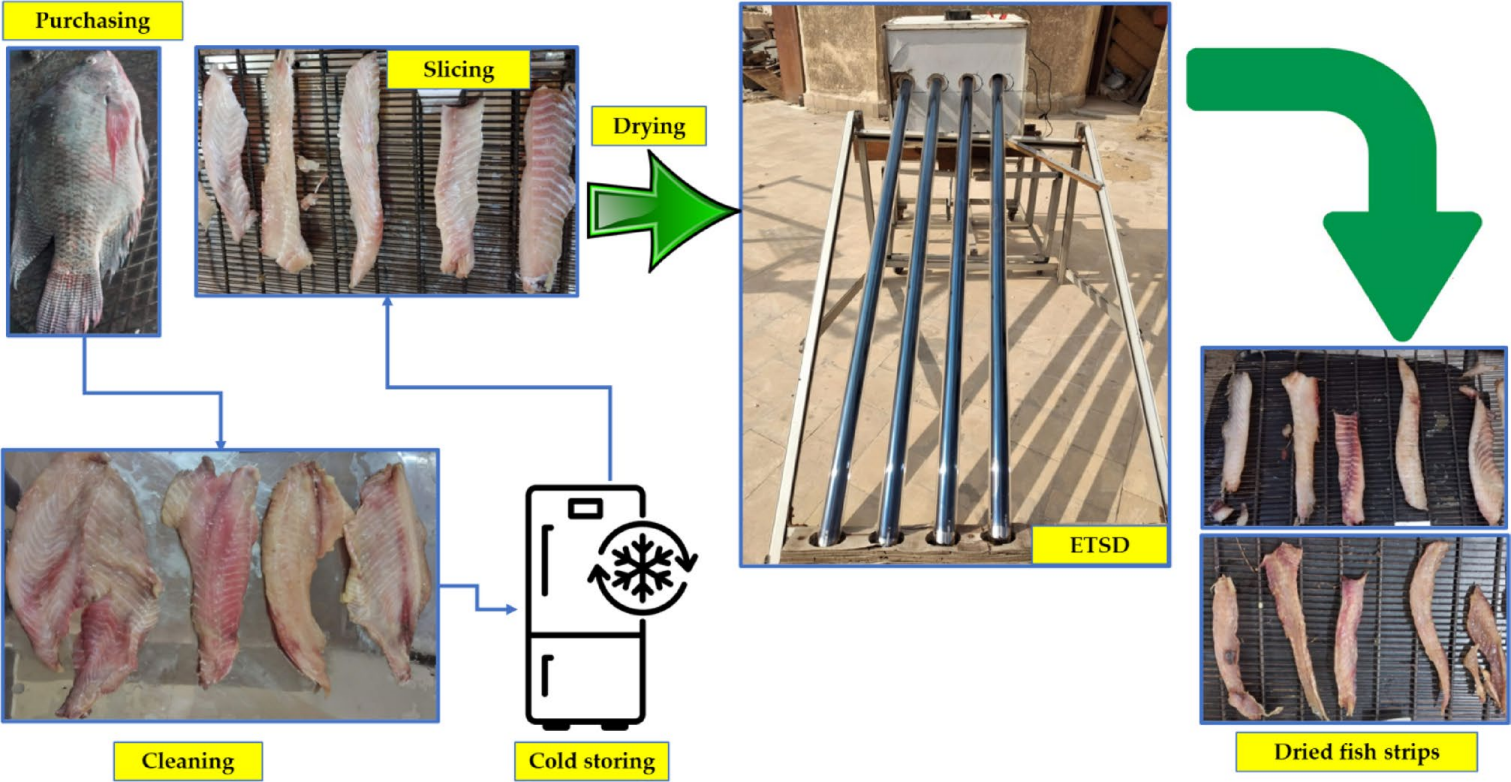
Step-by-step drying process flow diagram using the developed ETISD.

**Fig. 3:**
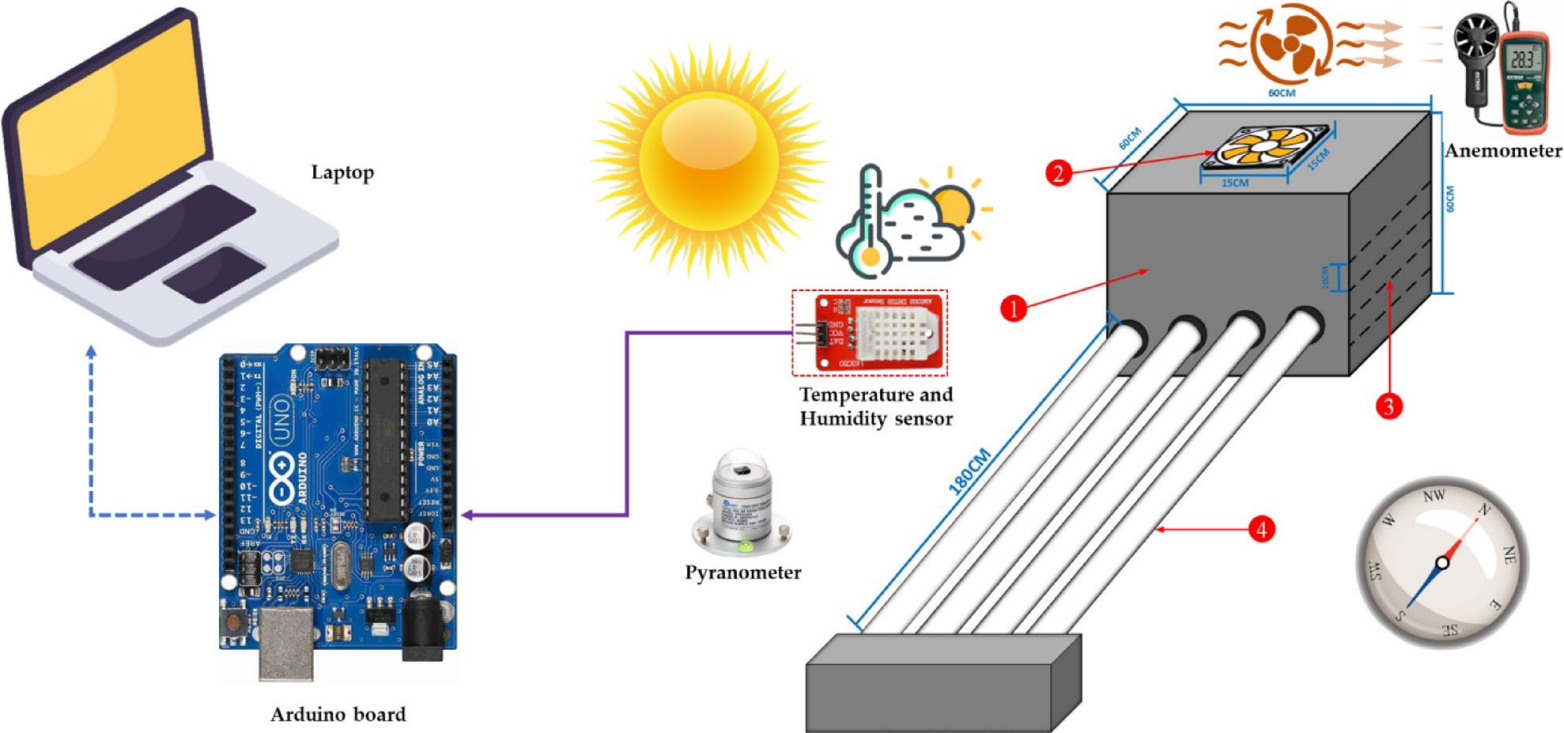
Schematic illustration of the experimental setup connected with measuring instrumentation, whereas (1) drying room, (2) exhaust fan, (3) drying trays, and (4) evacuated tubes.

All drying experiments were performed at the Agricultural Research Center in Giza, Egypt, in 2024. Figure 2 shows a step-by-step drying process flow diagram using the developed ETISD. The precooling tilapia fish was purchased from a local market in Cairo, Egypt. The initial moisture content of the tilapia fish samples was about 74.83% (w.b.) and 297.3% (d.b.). Then the fish were cleaned, filleted, and cut into slices at thicknesses of 4 mm, 8 mm, and 12 mm, with a thickness of 10 mm. Figure 3 illustrates a schematic illustration of the experimental setup connected with measuring instrumentation. The drying processes were conducted during the summer of 2024, from 8 a.m. to 5 p.m. During the drying processes, each sample’s weight, air temperature, air relative humidity, and SRI were measured hourly. Where the SRI was measured using an SRI sensor (model: SENTEC RS485, Sichuan, China), and both temperature and humidity of air were measured using a humidity and temperature sensor (model: DHT-22, China).

### Performance analysis

#### Moisture content (MC)

The initial MC of tilapia strips were conducted at 70 °C in an electric oven until reaching constant weight^63^. The MC of the tilapia samples was determined using Eq. (1)^64^.

1 $$MC \left( {w.b.} \right) = \frac{{\overbrace {{{\text{W}}_{{\text{w}}} - {\text{ W}}_{{\text{d}}} }}^{{\text{Difference between wet and dry weights of the tilapia sample }}}}}{{\overbrace {{{\text{W}}_{w} }}^{{\text{Weight of wet tilapia sample }}}}} \times 100$$

#### Energy analysis of the developed ETISD

Both ETSC and DR were assessed utilizing fundamental thermodynamic concepts of mass and energy conservation in steady-flow systems^65^. In accordance with the laws of mass conservation and energy, the air mass flow rate remains invariant throughout the ETISD, indicating that the inflow rate at the inlet exactly corresponds to the outflow rate at the exit.

2 $$\sum {\overbrace {{\dot{m}_{ai} }}^{Inlet mass flow rate}} = \sum {\overbrace {{\dot{m}_{ao} }}^{Outlet mass flow rate}}$$

3 $$\sum {\overbrace {{\dot{E}_{ai} }}^{Inlet energy flow rate}} = \sum {\overbrace {{\dot{E}_{ao} }}^{Outlet energy flow rate}}$$

4 $$\overbrace {{\dot{Q}}}^{Heat transfer } + \sum \dot{m}_{ai} \left( {\overbrace {{h_{ai} }}^{Inthalpy } + \frac{{\overbrace {{v_{ai} }}^{Velcity }}}{2} + \overbrace {{z_{ai} }}^{Height }g} \right) = \sum \dot{m}_{ao} \left( {\overbrace {{h_{ao} }}^{Inthalpy } + \frac{{\overbrace {{v_{ao} }}^{Velcity }}}{2} + \overbrace {{z_{ao} }}^{Height }g} \right) + \overbrace {{\dot{W}}}^{Work done}$$

where:

5 $$\overbrace {{\dot{W}}}^{{\text{Work done}}} = zero$$

6 $$\left[ {\frac{{\overbrace {{v_{ai} }}^{{\text{Velcity of input air }}}}}{2} - \frac{{\overbrace {{v_{ao} }}^{{\text{Velcity of output air }}}}}{2}} \right]\& \left[ {\overbrace {{z_{ai} }}^{{\text{Height of input air }}}g - \overbrace {{z_{ao} }}^{{\text{Height of output air }}}g} \right] = {\text{very small and it is neglected}}$$

#### Energy analysis of the ETSC and the developed ETSD

By applying Eqs. (5 and 6) to ETSC, the following equations were obtained.

7 $$\sum {\overbrace {{\dot{m}_{ai} }}^{Inlet mass flow rate}} = \sum {\overbrace {{\dot{m}_{ao} }}^{Outlet mass flow rate}} = \sum {\overbrace {{\dot{m}_{a} }}^{Mass flow rate}}$$

8 $$\overbrace {{\dot{Q}}}^{Heat trnsfer } = \overbrace {{\dot{Q}_{u, ETSC} }}^{Useful energy } = \overbrace {{\dot{Q}_{in, ETSC} }}^{Input energy } - \overbrace {{\dot{Q}_{ls, ETSC} }}^{Energy loss} = \overbrace {{\dot{m}_{a} }}^{Air mass flow rate}\left( {\overbrace {{h_{ao} - h_{ai} }}^{Change in enthalpy}} \right)$$

The input energy (${\dot{Q}}_{in, \text{ETSC}}$), useful energy (${\dot{Q}}_{u, \text{ETSC}}$) and energy efficiency (${\eta }_{en, \text{ETSC}}$) of the ETSC was calculated according to Eqs. 9–11^50,52,66^,

9 $$\overbrace {{\dot{Q}_{in, ETSC} }}^{Input energy } = \overbrace {{I_{s} }}^{Solar radation intensity } \times \overbrace {{A_{ETSC} }}^{Surface area of the ETSC }$$

10 $$\overbrace {{\dot{Q}_{u, ETSC} }}^{Useful energy } = \overbrace {{\dot{m}_{a} }}^{Mass flow rate} \times \overbrace {{C_{pa} }}^{Specific heat of air} \times \left( {\overbrace {{T_{co} - T_{ci} }}^{Change in air temperature}} \right)$$

11 $$\eta_{en, ETSC} = \frac{{\dot{Q}_{u, ETSC} }}{{\dot{Q}_{in, ETSC} }} = \frac{{\dot{m}_{a} C_{pa} \left( {T_{co} - T_{ci} } \right)}}{{I_{s} A_{ETSC} }}$$

The drying efficiency of the developed ETISD (${\eta }_{en, ETISD}$) was established using Eq. (12)^50^,

12 $$\eta_{en, ETISD} = \frac{{\overbrace {{m_{w} }}^{{\text{Quantity of remved water from fish sample}}} \times \overbrace {L}^{{\text{Latent heat of vaporization of water}}}}}{{\overbrace {{\dot{Q}_{{u, {\text{ETSC}}}} }}^{{\text{Useful energy }}} \times \overbrace {{t_{d} }}^{{\text{Drying time }}}}}$$

#### Exergy analysis ($Ex$)

The $\dot{E}x$ analysis of the developed ETISD is based on the second law of thermodynamics, and it is calculated using Eq. 13.

13 $$\begin{aligned} \overbrace {{\dot{E}x}}^{{\text{Exergy }}} = & \left( {\overbrace {{u - u_{\infty } }}^{{\text{Internal energy}}}} \right) - T_{0} \left( {\overbrace {{s - s_{\infty } }}^{{E{\text{ntropy}}}}} \right) + P_{0} \left( {\overbrace {{v - v_{\infty } }}^{{F{\text{low work}}}}} \right) + \frac{{\overbrace {{V^{2} }}^{{M{\text{omentum energy}}}}}}{2} \\ \quad & + g\left( {\overbrace {{z - z_{\infty } }}^{{G{\text{ravitational energy}}}}} \right) + \mathop \sum \limits_{ch} \left( {\overbrace {{\mu_{ch} - \mu_{\infty } }}^{{\text{Chemical energy }}}} \right) \\ \quad & \times N_{ch} + \left( {\overbrace {{\sigma A_{i} F_{i} \left( {3T^{4} - T_{\infty }^{4} - 4T_{\infty } T^{3} } \right)}}^{{\text{Radiation energy}}}} \right) \\ \end{aligned}$$

By applying the Eq. (13) in the current study for the developed ETISD, it will be rewritten by neglecting the unnecessary parts related to the flow process. The momentum, gravitational, chemical and radiation energies. And Eq. (14) by applying the above assumptions^47^.

14 $$\dot{E}x = \dot{m}_{a} C_{pa} \left( {\left( {\overbrace {{T - T_{0} }}^{Change in air temperature}} \right) - T_{0} ln\left( {\frac{T}{{T_{0} }}} \right)} \right)$$

where, ${T}_{0}$ is atmospheric air temperature.

##### Exergy analysis of the ETSC

$\dot{E}x$ Balance for ETSC is given by Eq. (19)^50,67,68^,

15 $$\overbrace {{\dot{E}x_{ls, ETSC} }}^{Exergy loss } = \overbrace {{\dot{E}x_{in, ETSC} }}^{Input exergy } - \overbrace {{\dot{E}x_{out, ETSC} }}^{Output exergy}$$

16 $$\dot{E}x_{in, ETSC} = \left[ {1 - \frac{{\overbrace {{T_{0} }}^{Ambient air temperature }}}{{\overbrace {{T_{s} }}^{{Sun temperature \left( {6000 k} \right) }}}}} \right] \times \overbrace {{\dot{Q}_{in, abs} }}^{Energy absorbed by the absorper plate }$$

17 $$\dot{Q}_{in, abs} = \overbrace {\tau }^{{Transmissivity\; of\;glass \left( {0.88} \right) }} \times \overbrace {\alpha }^{{Absorptivity\; of\;glass \left( {0.95} \right) }} \times \dot{Q}_{in, ETSC}$$

18 $$\dot{E}x_{out, ETSC} = \dot{m}_{a} C_{pa} \left( {\left( {T_{co} - T_{ci} } \right) - T_{0} ln\left( {\frac{{T_{co} }}{{T_{ci} }}} \right)} \right)$$

The $\dot{E}x$ efficiency of ETSC is obtained using Eq. ^50,69^,

19 $$\eta_{ex, ETSC} = \frac{{\dot{E}x_{out, ETSC} }}{{\dot{E}x_{in, ETSC} }} = 1 - \frac{{\dot{E}x_{ls, ETSC} }}{{\dot{E}x_{in, ETSC} }} = 1 - \frac{{T_{0} S_{gen} }}{{\left[ {1 - \frac{{T_{0} }}{{T_{s} }}} \right]\dot{Q}_{in, ETSC} }}$$

##### $\dot{E}x$ analysis of the DR

$\dot{E}x$ balance for DR is expressed as follows:

20 $$\overbrace {{\dot{E}x_{ls, DR} }}^{Exergy loss } = \overbrace {{\dot{E}x_{in, DR} }}^{Input exergy } - \overbrace {{\dot{E}x_{out,DR} }}^{Output exergy}$$

The ${\dot{E}x}_{in, DR}$, ${\dot{E}x}_{out,DR}$ and exergy efficiency (${\eta }_{ex, DR}$) of the DR are calculated using Eqs. (21–23)^47,70^,

21 $$\dot{E}x_{in, DR} = \dot{m}_{a} C_{pa} \left( {\left( {T_{in,DR} - T_{0} } \right) - T_{0} ln\left( {\frac{{T_{in,DR} }}{{T_{0} }}} \right)} \right)$$

22 $$\dot{E}x_{out, DR} = \dot{m}_{a} C_{pa} \left( {\left( {T_{out,DR} - T_{ci} } \right) - T_{0} ln\left( {\frac{{T_{out,DR} }}{{T_{0} }}} \right)} \right)$$

where, ${T}_{in,DR}$ and ${T}_{out,DR}$ are inlet and outlet air temperatures from the DR, respectively.

23 $$\eta_{ex, DR} = \frac{{\dot{E}x_{out, DR} }}{{\dot{E}x_{in, DR} }}$$

#### Sustainability indicators

During the current study, three $\dot{E}x$ sustainability indicators were estimated according to the following Equation from 24 to 26^47^.

24 $$provement potential \left( {IP} \right) = \left( {1 - \eta_{ex} } \right)\dot{E}x_{ls}$$

25 $$Waste \;exergy\; ratio \left( {WER} \right) = \frac{{\dot{E}x_{ls} }}{{\dot{E}x_{in} }}$$

26 $$Sustainability index \left( {SI} \right) = \frac{1}{{1 - \eta_{ex} }}$$

### CFD simulation setup

In this study, the CFD simulation was conducted under transient conditions to more accurately capture the dynamic behavior of the drying process. This choice was made to account for the time-dependent variations in solar radiation, airflow distribution, and the operation of exhaust fans, all of which significantly influence the drying kinetics. Transient analysis provides a more realistic representation of the unsteady heat and mass transfer phenomena occurring during drying, especially in solar-assisted systems where environmental inputs fluctuate throughout the day. The implementation of CFD generally involves three main stages: (i) Preprocessing – This phase includes defining the geometry of the computational domain, which represents the system or region where fluid flow is to be modeled. The domain is then divided into numerous discrete elements, forming a computational mesh. At this stage, boundary conditions, physical models, material and fluid properties, and the numerical parameters required for solving the equations are also established. (ii) Processing – The simulation is carried out using iterative numerical calculations. (iii) Post-processing – The results are analyzed and visualized using various graphical representations such as profiles, contour plots, streamlines, and surfaces, illustrating variables like velocity, pressure, temperature, and concentration^26^.

#### Governing equations

The governing equations for mass, momentum, and energy conservation, which are solved using CFD in this study, are shown in Eqs. 27–29.

27 $${\text{Mass }}\;{\text{conservation }}\;{\text{equation}}:\nabla \cdot \left( {pv} \right) = 0$$

28 $${\text{Momentum }}\;{\text{conservation }}\;{\text{equation}}:\nabla \cdot \left( {pv} \right) = \nabla \cdot \left( {\mu \nabla v} \right) - \nabla P + \rho g$$

29 $${\text{Energy }}\;{\text{conservation}}\;{\text{ equation}}:\nabla \cdot \left( {\vec{v}\left( {\rho E + P} \right)} \right) = - \nabla \cdot \left( {\mathop \sum \limits_{j} h_{j} J_{j} } \right) + S_{h}$$

The air flow in the greenhouse dryer was turbulent; therefore, the mean flow variables must be solved in conjunction with a turbulence model that describes the effect of turbulence on the mean flow. In this study, the standard k-ε turbulence model integrated in SolidWorks 2019 was used, because it provides convergence in a short time and accurate results. The standard k-ε model is a semiempirical model that contains and solves two equations, the first for the turbulent kinetic energy (*k*) and the second for the dissipation rate of this energy (*ε*), which are based on the Reynolds equation and Navier–Stokes. The developed equations are shown below:

Turbulent kinetic energy:

30 $$\frac{\partial }{{\partial x_{i} }}\left( {\rho kv_{i} } \right) = \frac{\partial }{{\partial x_{j} }}\left[ {\left( {\mu + \frac{{\mu_{t} }}{{\sigma_{k} }}} \right)\frac{\partial k}{{\partial x_{j} }}} \right] + P_{k} + P_{b} - \rho_{\varepsilon } - Y_{M} + S_{k}$$

Turbulent dissipation rate:

31 $$\frac{\partial }{{\partial x_{i} }}\left( {\rho \in v_{i} } \right) = \frac{\partial }{{\partial x_{j} }}\left[ {\left( {\mu + \frac{{\mu_{t} }}{{\sigma_{\varepsilon } }}} \right)\frac{\partial \varepsilon }{{\partial x_{j} }}} \right] + \rho C_{1} S_{\varepsilon } - \rho C_{2} \left( {\frac{{\varepsilon^{2} }}{{k + \sqrt {v\varepsilon } }}} \right) + C_{1\varepsilon } \frac{\varepsilon }{k}C_{3\varepsilon } P_{b} + S_{\varepsilon }$$

where, $${\sigma }_{k}=1.0$$; $${\sigma }_{\varepsilon }=1.2;$$ $${C}_{1\varepsilon }=1.44$$; $${C}_{2}=1.9$$

32 $$C_{1} = max\left[ {0.43,\frac{\eta }{\eta + 5}} \right]$$

33 $$\eta = S\frac{k}{\varepsilon }$$

34 $$S = \sqrt {2S_{ij} S_{ij} }$$

In the turbulent viscosity, we have

35 $$\mu_{t} = \rho \mu_{\mu } \frac{{k^{2} }}{\varepsilon }$$

36 $$C_{t} = \frac{1}{{A_{o} + A_{S} \frac{{kU^{*} }}{\varepsilon }}}$$

37 $$U^{*} = \sqrt {S_{ij} S_{ij} + \tilde{\Omega }_{ij} \tilde{\Omega }_{ij} }$$

38 $$\tilde{\Omega }_{ij} = \Omega_{ij} - 2 \in_{ijk} \omega_{k}$$

39 $$\Omega_{ij} = \overline{\Omega }_{ij} - \in_{ijk} \omega_{k}$$

where, ${\overline{\Omega } }_{ij}$ is the tensor of the center of rotation velocity in a rotating zone with angular velocity ${\Omega }_{k}$. They have constants ${A}_{o}$ and ${A}_{S}$ that are described by Eqs. 40–45;

40 $$A_{o} = 4.04$$

41 $$A_{S} = \sqrt 6 cos\varphi$$

42 $$\varphi = \frac{1}{3}cos^{ - 1} \left( {\sqrt 6 W} \right)$$

43 $$W = \frac{{S_{ij} S_{ik} S_{ki} }}{{\tilde{S}^{3} }}$$

44 $$\tilde{S} = \sqrt {S_{ij} S_{ij} }$$

45 $$S_{ij} = \frac{1}{2}\left( {\frac{{\partial u_{j} }}{{\partial x_{i} }} + \frac{{\partial u_{i} }}{{\partial u_{j} }}} \right)$$

#### Boundary conditions

The values of the boundary conditions used in this study are the averages of the experimental measurements carried out with the experimental equipment during the current study. The numerical finite volume method, as used in (SolidWorks 2019), is used for solving the equations on a PC P 13^th^ Gen Intel(R) Core (TM) i7-13620H 2.40 GHz with 16 GB RAM. In this study, various boundary conditions are defined as follows:

- Inlet: inflow condition is given. Environmental pressure = 101325 Pa, air temperature of 40.1 °C. The software extrapolates the required information from the interior of the drying chamber to the outlet.
- Outlet: outlet five air flow rates of 0.01, 0.02, 0.03, 0.04, and 0.05 m^3^/s.
- Porous media: empirical parameters of pressure drop equation and leaves tray porosity are defined.
- Gravitational acceleration (m = s^2^) 9.8
- Bed wall boundary condition: Bed wall boundary condition
- Basic mesh dimensions: 12 × 24 × 48
- Analysis mesh: total cell count: 43645; fluid cells:17361; solid cells: 26284; partial cells: 9815

### Uncertainty analysis

The measurement uncertainties for critical drying parameters were quantified using Eq. 46, yielding values of 0.32% for temperature, 0.28% for relative humidity, 0.24% for wind speed, and 0.13% for solar radiation. Propagating these individual uncertainties through the system efficiency calculations resulted in a combined uncertainty of ± 2% for the overall dryer performance evaluation.

46 $${\mathcal{W}}_{r} = \left[ {\left( {\frac{\partial R}{{\partial x_{1} }}{\mathcal{W}}_{1} } \right)^{2} + \left( {\frac{\partial R}{{\partial x_{2} }}{\mathcal{W}}_{2} } \right)^{2} + \cdots + \left( {\frac{\partial R}{{\partial x_{3} }}{\mathcal{W}}_{3} } \right)^{2} } \right]^{1/2}$$

## Results and discussion

### Moisture content (MC)

**Fig. 4:**
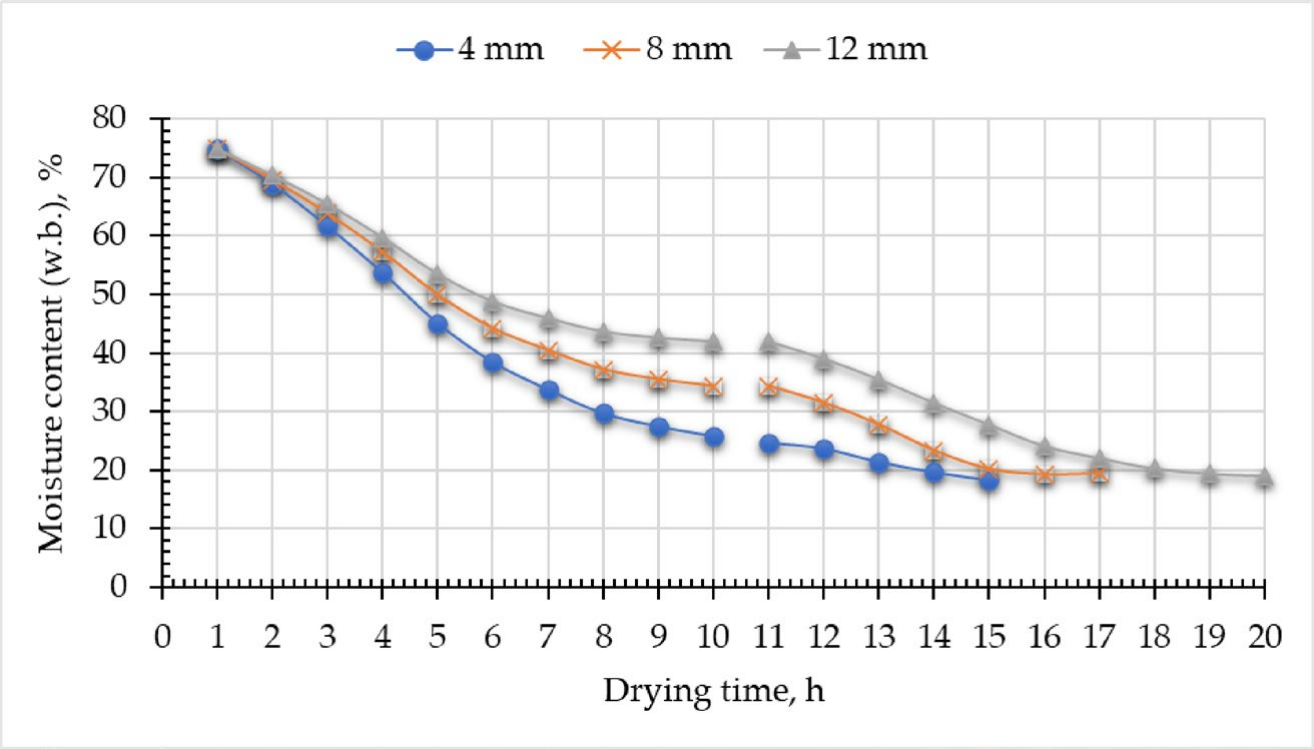
MC of tilapia strips dried using the developed ETISD.

The variation in moisture content at different strip thicknesses during the experiment is graphically depicted in Fig. 4. The initial MC was about 74.83% (w.b.), which subsequently diminished by 18.84%, 18.8%, and 19.45% after 15, 17, and 20 h at slice thicknesses of 4 mm, 8 mm, and 12 mm, respectively, for different samples dried utilizing ETISD.

### Using CFD to estimate the appropriate air speed of the exhaust fan

Computational Fluid Dynamics (CFD) is a powerful numerical tool for optimizing the airflow in solar drying systems by simulating fluid behavior under varying conditions. In SDs, selecting the appropriate exhaust fan speed is critical to balancing efficient moisture removal and energy consumption. CFD enables precise analysis of air velocity, temperature distribution, and pressure gradients within the drying chamber, eliminating the need for costly experimental trials. By modeling different fan speeds, CFD helps identify the optimal airflow rate that ensures uniform drying while minimizing heat loss^27,30,71–73^. During the current study, the CFD was conducted using SolidWorks software version 2019.

**Fig. 5:**
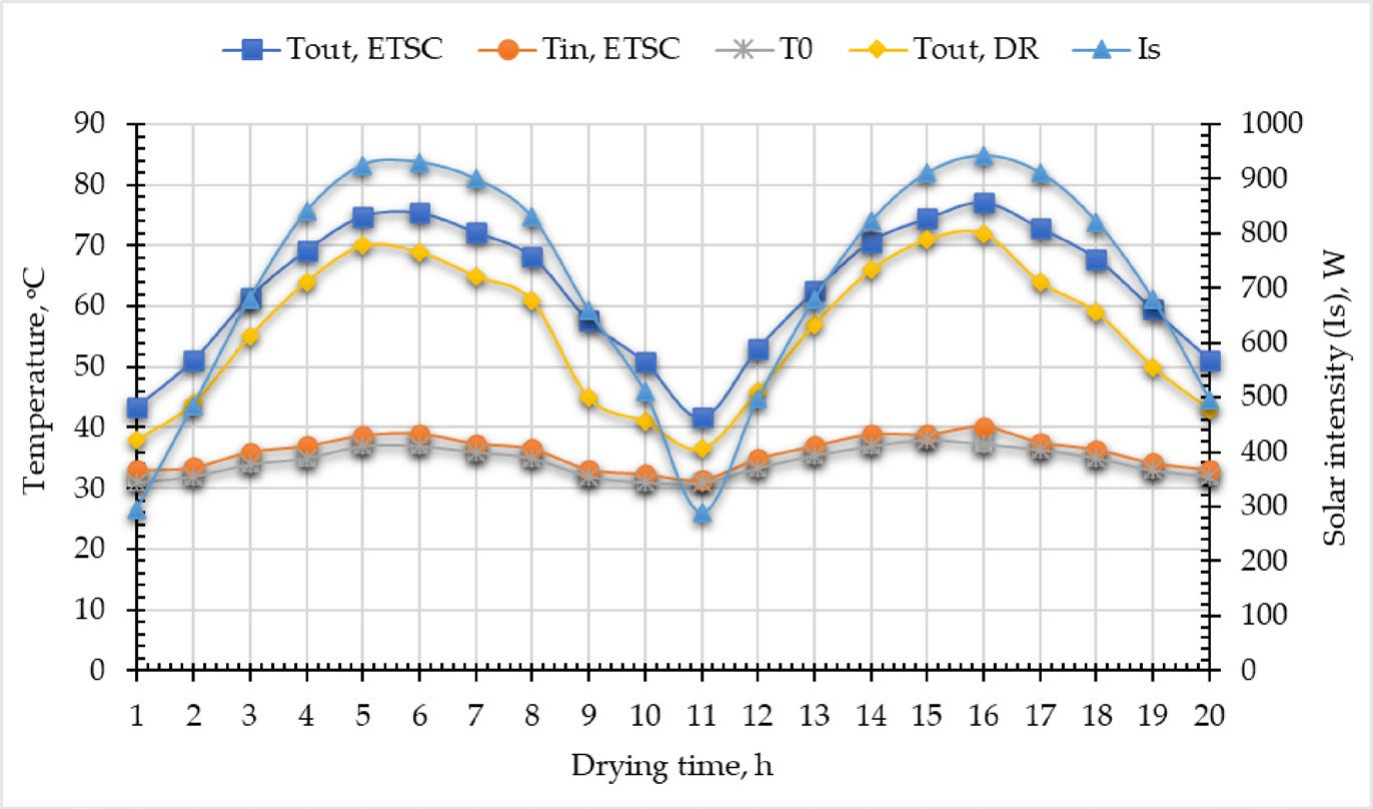
Recorded and predicted air temperature and SRI during drying process.

Figure 5 illustrates recorded air temperature and SRI using the DHT-22 sensor and SENTEC RS485 sensor, as well as the predicted inlet and outlet air temperature from the developed ETISD during the drying process throughout the 2 days of field tests from 8 a.m. to 5 p.m. for 10 h per day (24 and 25 June 2024). The presented data in the same figure showed that SRI gradually increased, peaking at approximately 925 W/m^2^ and 911 W/m^2^ for both drying days at 1 p.m. The ambient air temperature followed a similar trend, reaching a maximum of 37 °C at 1 p.m. Also, Fig. 5 shows that the outlet air temperature from the SAC ranged between 41.69 and 74.82 °C at 8 a.m. and 1 p.m., respectively. While the outlet air temperature from the DR ranged between 38 and 70 °C at the time. All measured data of air temperature were conducted at a constant air flow rate of 0.03 m^3^/s, and the air mass flow rate ranged between 0.025 and 0.026 kg/s.

**Fig. 6:**
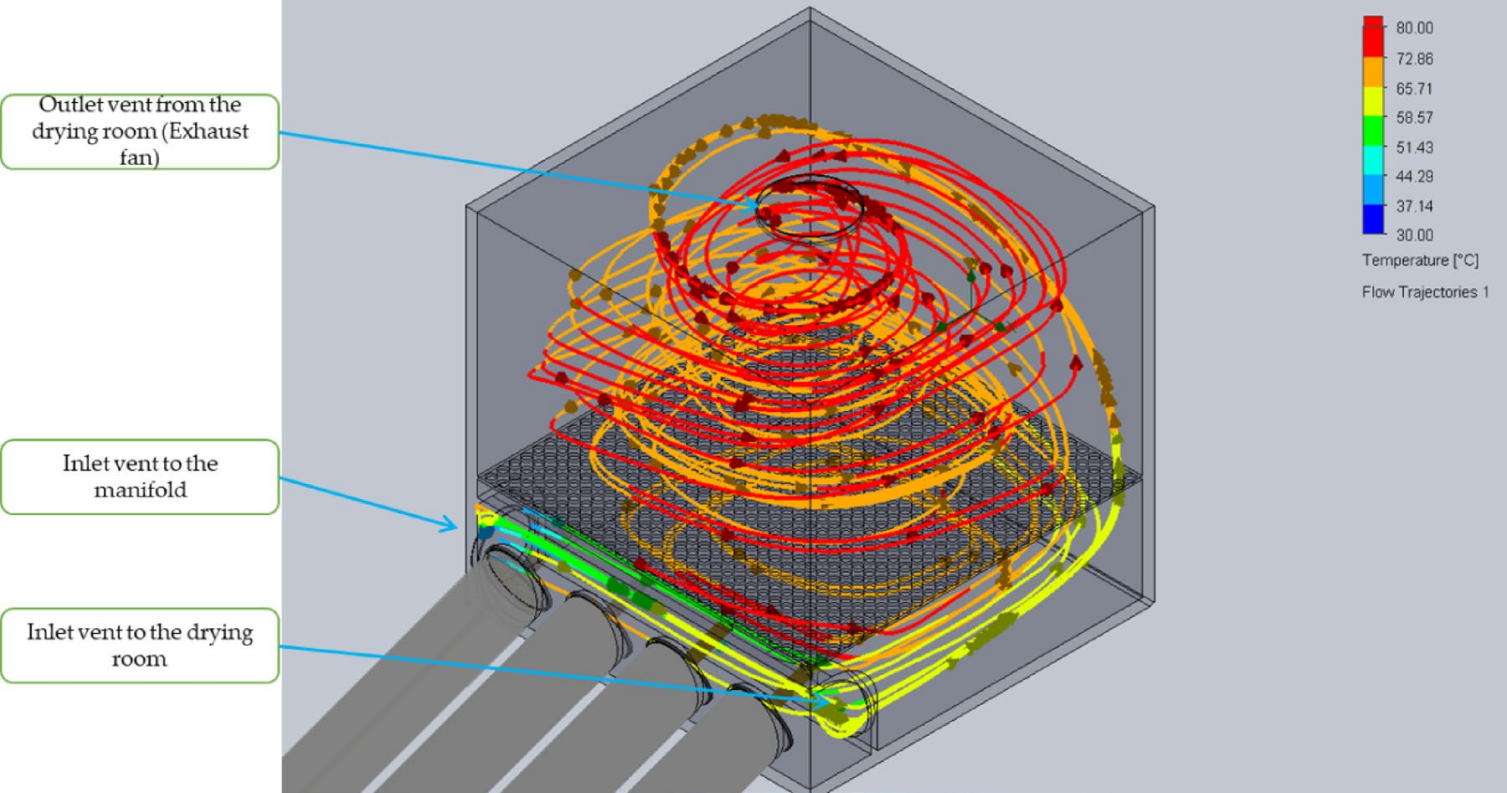
Air flow trajectories inside the manifold and the drying room.

Airflow path lines and velocity vectors inside the developed ETISD at solar noon (1 p.m.) are shown in Fig. 6. As can be seen from Fig. 6, fresh air enters through the inlet of the manifold at a temperature of 38.7 °C, which gradually increases up to 74.82 °C at the exhaust vent of the manifold while blowing over the heat pipes of the evacuated tubes, and then enters inside the DR and flows through the drying trays and finally exits from the chimney by the exhaust fan with an average temperature of 70 °C. The maximum temperature (air) inside the DR was shown to be 74.82 °C around solar noon (1 p.m.), which was about 4.82 °C more than the exhausted air by the chimney.

In the present study, five different air volume flow rates of 0.02, 0.03, 0.04, 0.05, and 0.06 m^3^/s, were evaluated to analyze their impact on the internal air temperature and velocity distribution within the DR. The primary objective was to determine the most suitable air velocity (air volume flow rate) that maintains optimal drying conditions, particularly the temperature required for high-quality drying of tilapia fish. CFD simulations were employed to visualize and assess the airflow behavior and temperature distribution within the DR under each air volume flow rate condition.

**Fig. 7:**
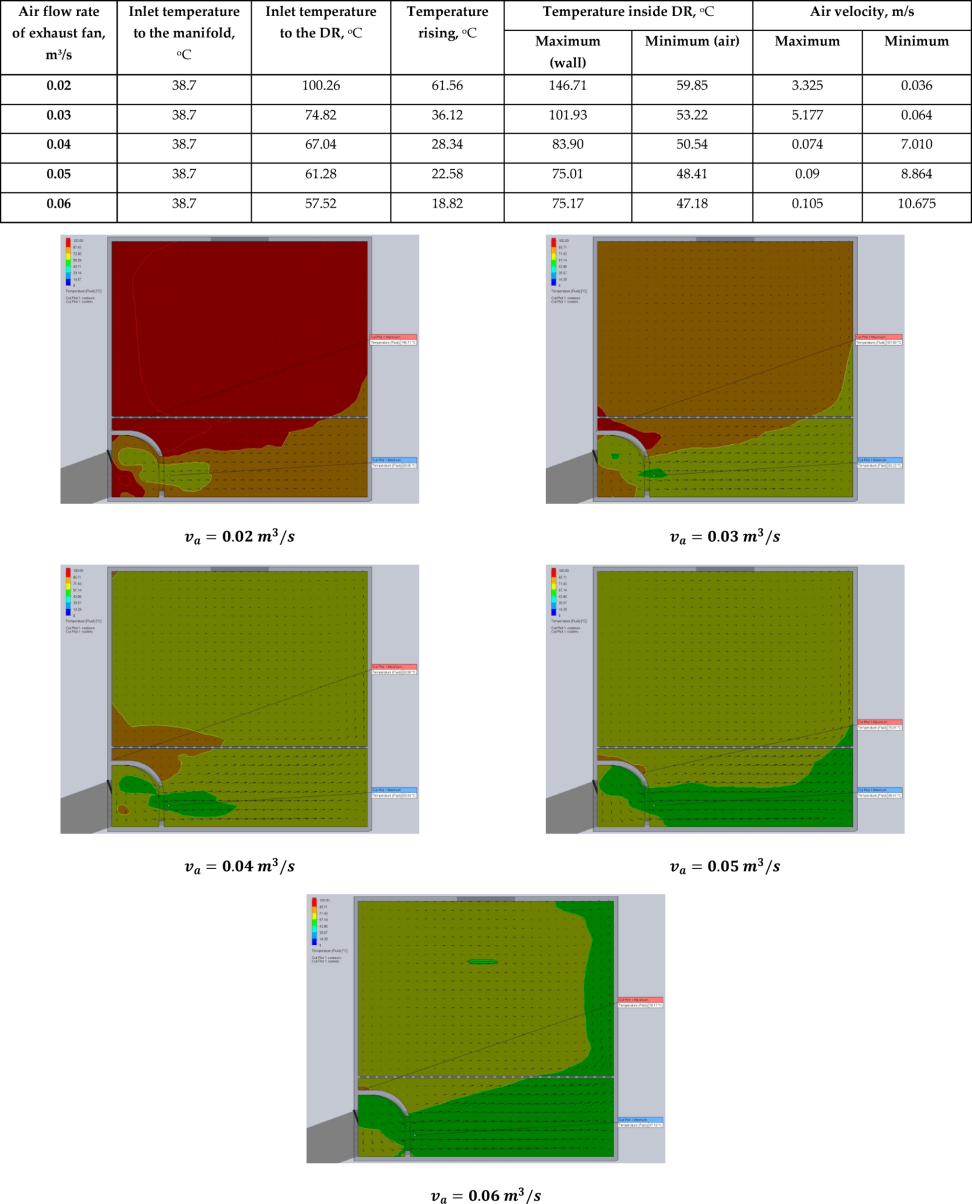
The contours of air temperatures inside the drying room at different air velocities of the exhaust fan (0.02, 0.03, 0.04, 0.05, and 0.06 m^3^/s).

**Fig. 8:**
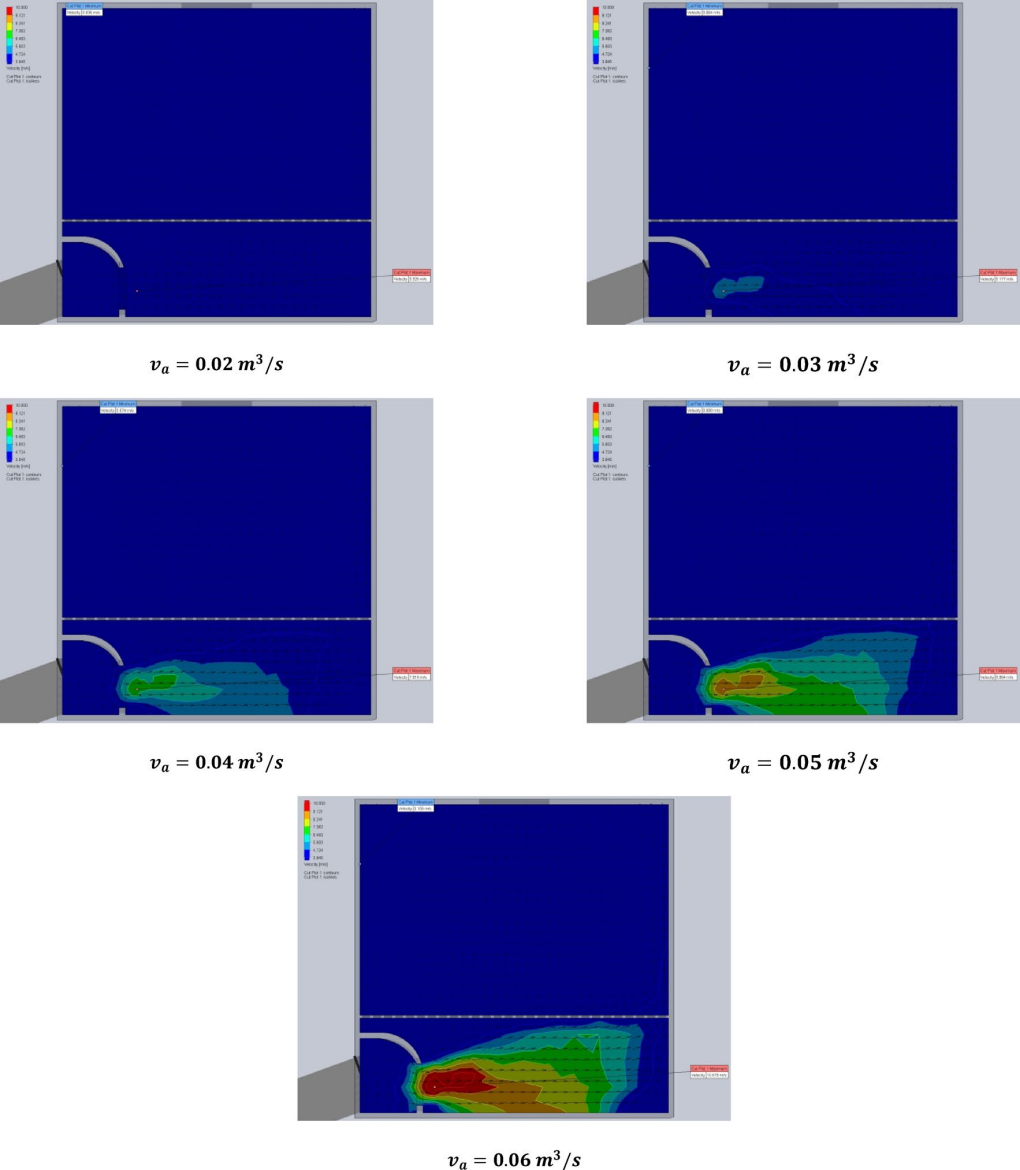
The contours of air velocity inside the drying room at different air velocities of the exhaust fan (0.02, 0.03, 0.04, 0.05, and 0.06 m^3^/s).

The simulation results are presented in Figs. 7 and 8. Specifically, Fig. 7 illustrates the air temperature contours, while Fig. 8 depicts the airflow velocity profiles within the DR at various exhaust fan settings. These visualizations highlight the influence of fan-induced airflow on the thermal environment inside the DR.

According to Sanda et al.^63^, the optimal drying temperature range for tilapia fish lies between 60 and 80 °C to ensure favorable product quality. Based on the data summarized in Table 1 and CFD analysis, an air flow rate of 0.03 m^3^/s (equivalent to a volumetric flow rate of 0.025 m^3^/s) was identified as the most effective setting. At this air volume flow rate, the temperature inside the DR reaches approximately 74.82 °C, at solar noon (around 1:00 p.m.), which falls within the ideal drying temperature range.

**Table 1:** The observed temperatures and air velocities inside the DR based on the obtained results of simulation using the CFD at solar noon.

Air flow rate of exhaust fan, m^3^/s	Inlet temperature to the manifold, °C	Inlet temperature to the DR, °C	Temperature rising, °C	Temperature inside DR, °C		Air velocity, m/s	
				Maximum (wall)	Minimum (air)	Maximum	Minimum
0.02	38.7	100.26	61.56	146.71	59.85	3.325	0.036
0.03	38.7	74.82	36.12	101.93	53.22	5.177	0.064
0.04	38.7	67.04	28.34	83.90	50.54	0.074	7.010
0.05	38.7	61.28	22.58	75.01	48.41	0.09	8.864
0.06	38.7	57.52	18.82	75.17	47.18	0.105	10.675

It was observed that increasing the air volume flow rate beyond 0.03 m/s resulted in a noticeable decrease in the internal temperature of the DR. This inverse relationship occurs because higher air velocities reduce the residence time of air in contact with the heated surfaces of the evacuated tube heat exchangers. As a result, less thermal energy is transferred to the airflow, leading to lower internal air temperatures. Therefore, balancing air volume flow rate is crucial—not only to ensure effective heat transfer but also to maintain the desired drying temperature without compromising energy efficiency or product quality.

### Using CFD to estimate the air velocity patterns and fluid temperature distribution inside the drying room

**Fig. 9:**
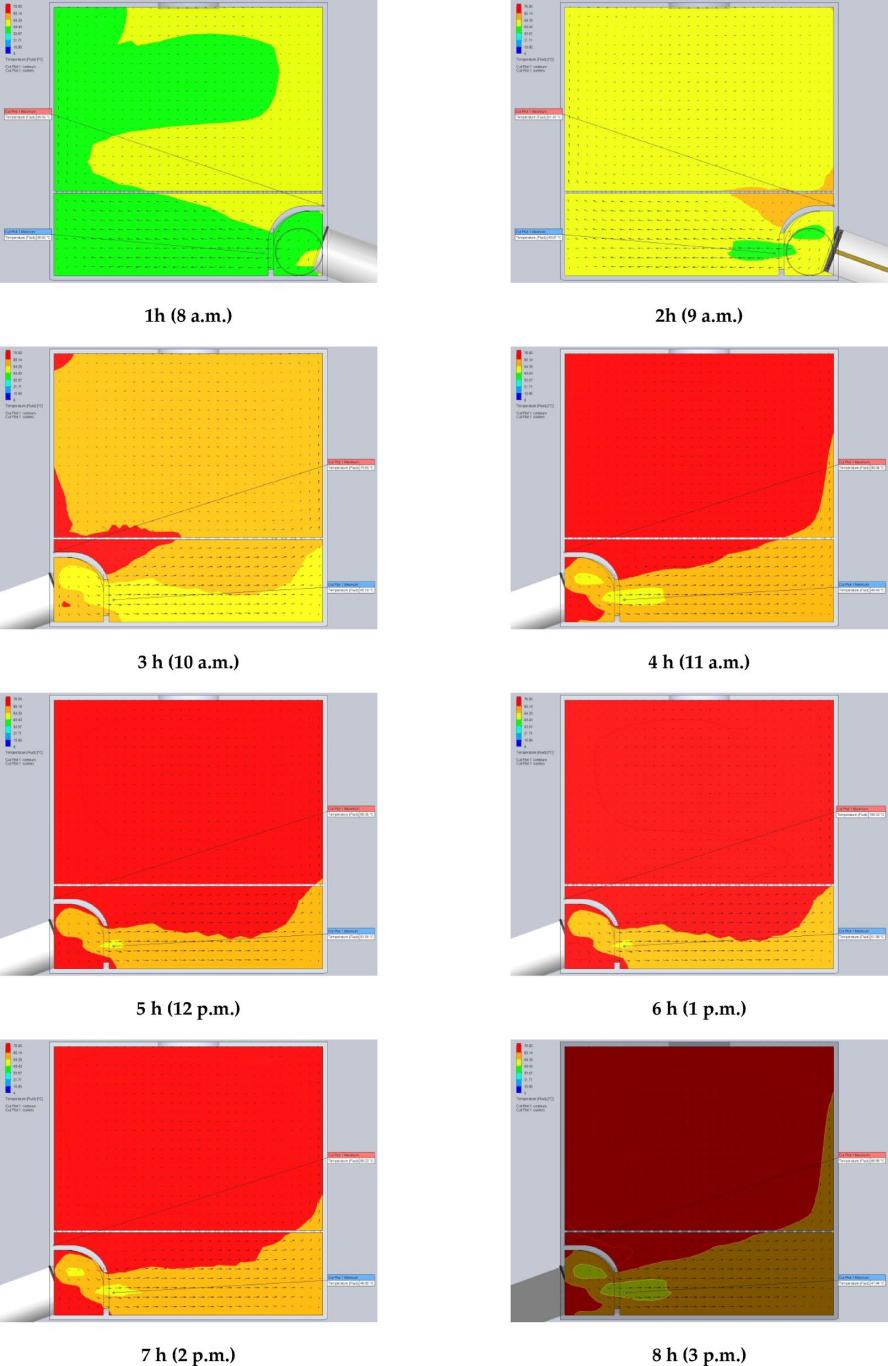

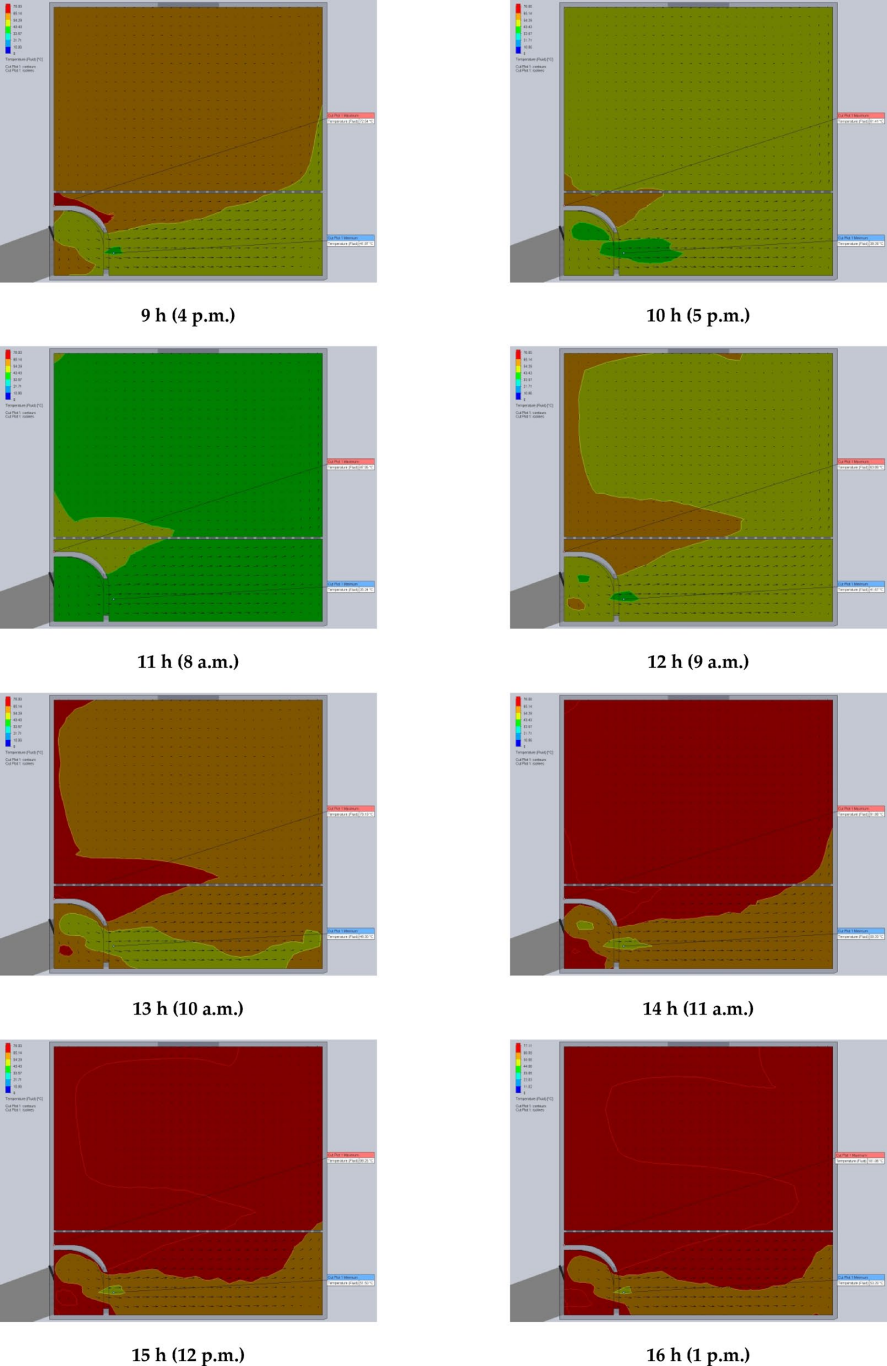

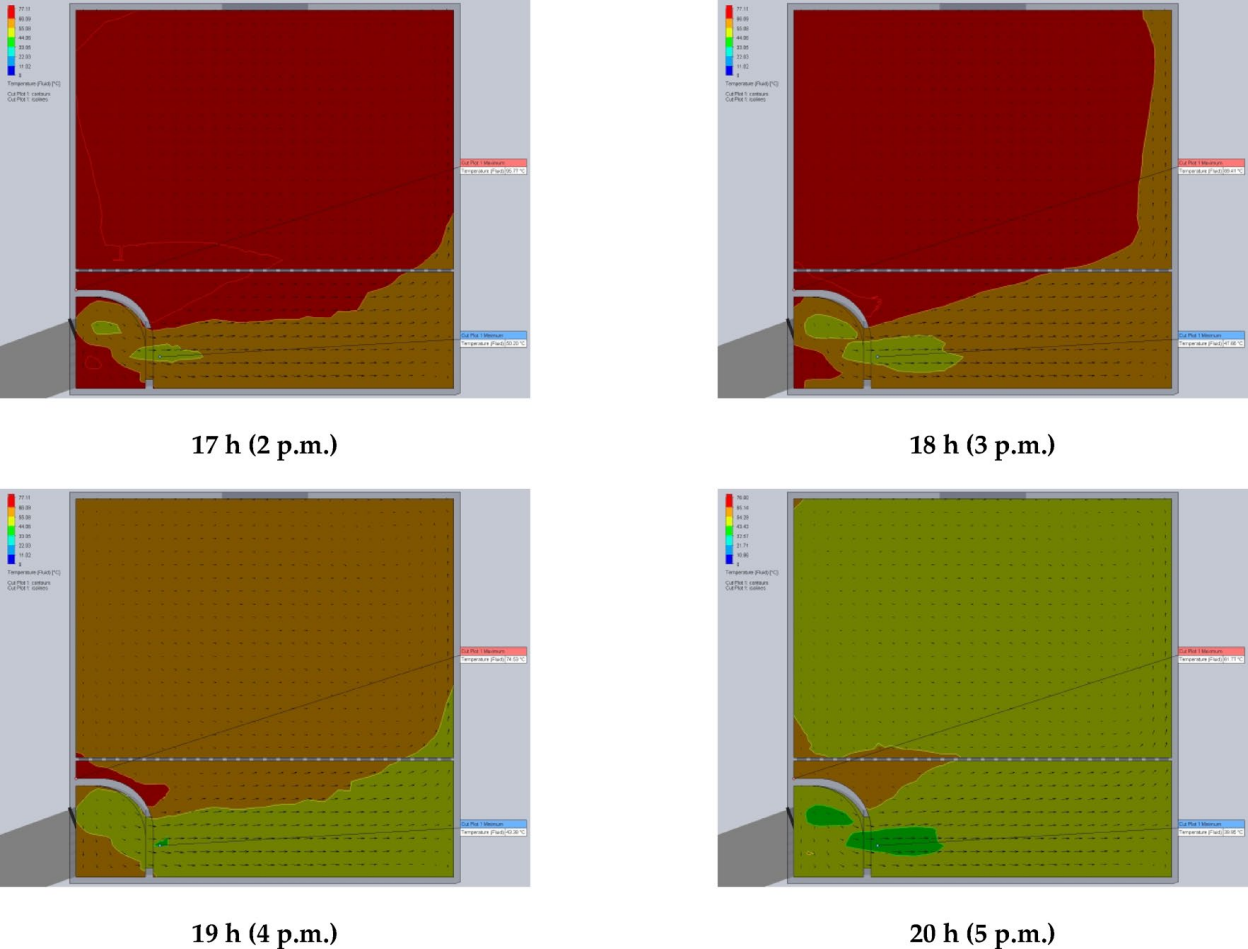
The contours of air temperatures inside the drying room throughout the two days of the drying experiments from 8 a.m. to 5 p.m.

**Fig. 10:**
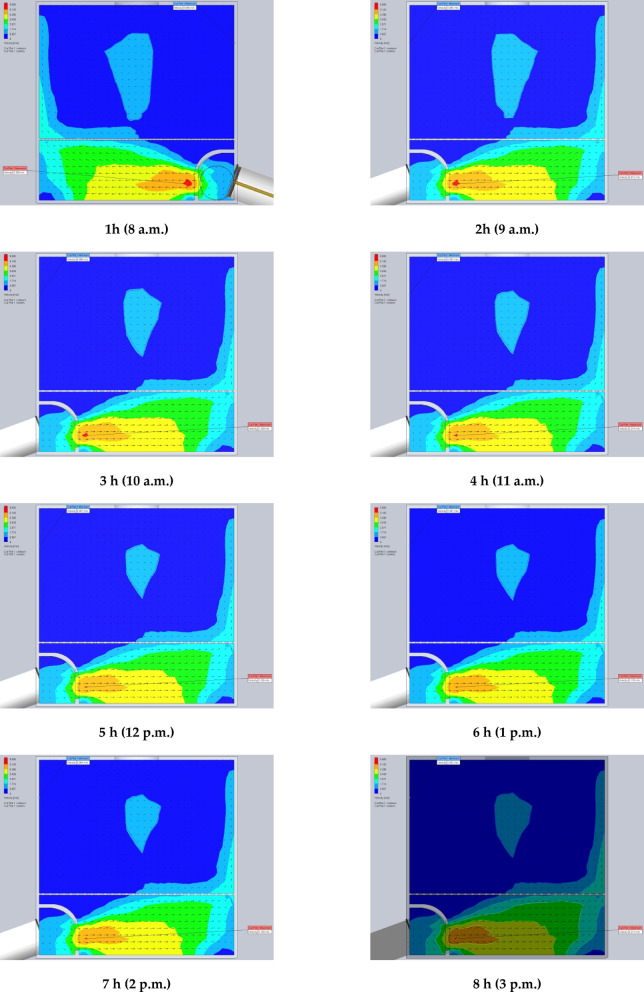

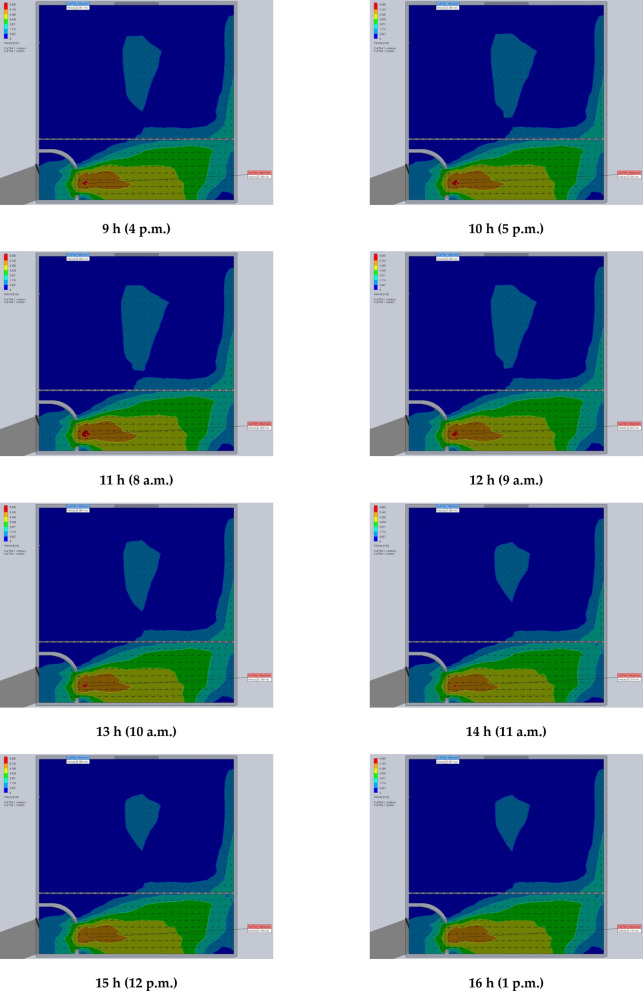

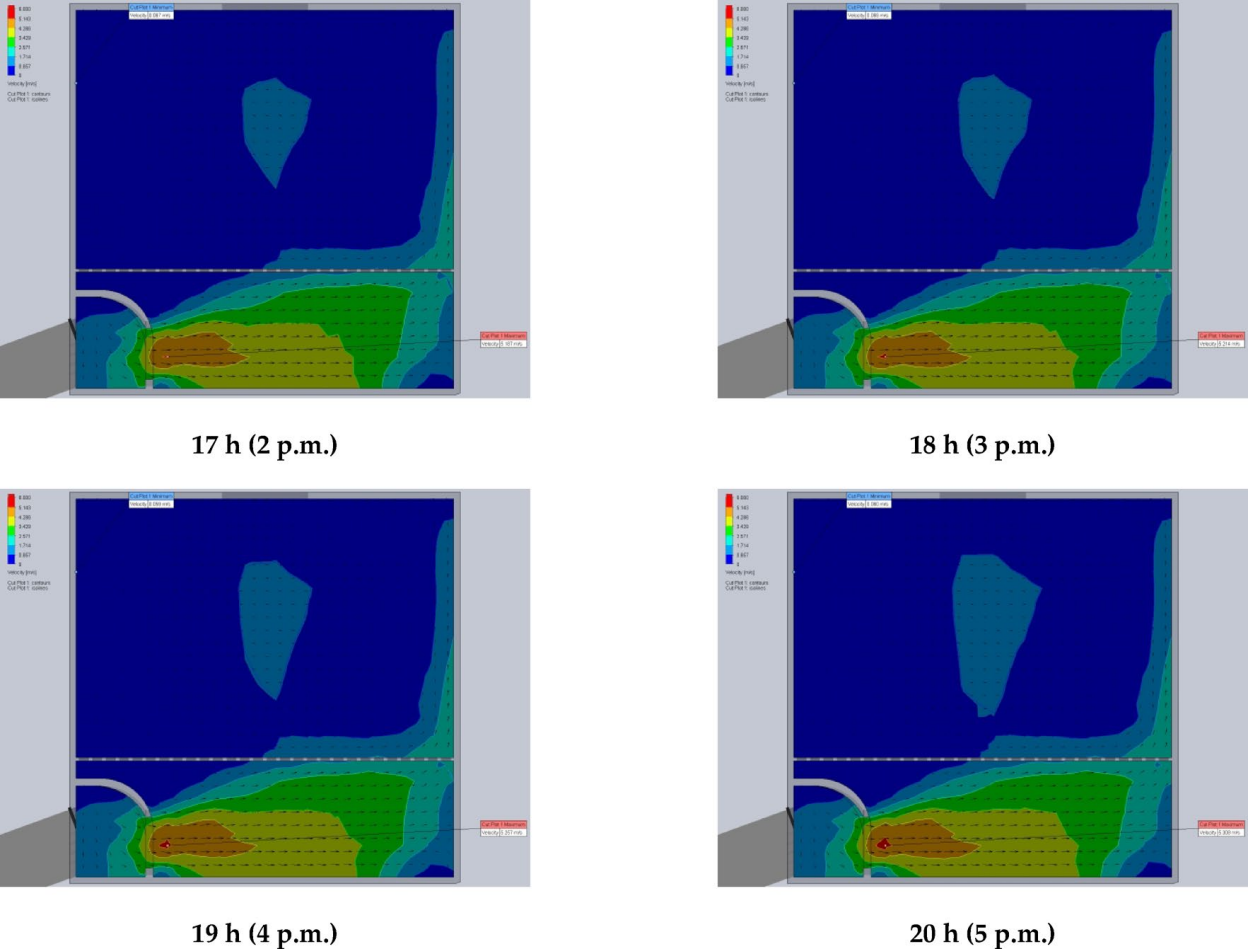
The contours of air velocity inside the drying room throughout the two days of the drying experiments from 8 a.m. to 5 p.m.

The time-variable boundary condition, including solar heat and ambient temperature was applied in the model based on the collected weather data from field experiments. The weather-based time-variable boundary conditions for 20 models (one model for every hour of two days) are shown in Fig. 9. The CFD simulations were carried out with the objective of analyzing and evaluating temperatures and air velocities inside the DR throughout the two days of the drying experiments from 8 a.m. to 5 p.m. The results helped in the study of the fluid dynamic behavior of the air inside the DR (Figs. 9 and 10). Table 2 shows the observed temperatures and air velocities inside the DR based on the obtained results of simulation using the CFD throughout the two days of the drying experiments from 8 a.m. to 5 p.m. As shown in Fig. 10 the airflow moves smoothly through the manifold before accelerating as it passes through the inlet vent into the DR. However, the porous drying trays—loaded with tilapia strips—act as a significant flow barrier, causing a drastic reduction in air velocity. Figure 10 clearly demonstrates this deceleration as the air passes through the trays. Once beyond the trays, the airflow regains speed before exiting through the chimney.

**Table 2:** The observed temperatures and air velocities inside the DR based on the obtained results of simulation using the CFD throughout the two days of the drying experiments from 8 a.m. to 5 p.m.

Drying time, h	Inlet temperature to the manifold, °C	Inlet temperature to the DR, °C	Temperature rising, °C	Temperature inside DR, °C		Air velocity, m/s	
				Maximum (wall)	Minimum (air)	Maximum	Minimum
1	33	43.44	10.44	49.55	36.82	5.369	0.060
2	33.5	51.13	17.63	61.47	40.07	5.312	0.060
3	36	61.53	25.53	78.60	45.18	5.259	0.060
4	37	69.19	32.19	90.04	48.49	5.120	0.063
5	38.7	74.82	36.12	98.35	51.54	5.185	0.067
6	39	75.4	36.4	100.33	51.98	5.176	0.067
7	37.4	72.23	34.83	96.22	49.95	5.190	0.064
8	36.5	68.22	31.72	88.96	47.94	5.212	0.062
9	33.2	57.7	24.48	72.54	41.97	5.266	0.061
10	32.4	50.83	18.43	61.41	39.28	5.305	0.059
11	31.5	41.69	10.19	47.95	35.25	5.369	0.058
12	34.9	53.06	18.16	63.89	41.67	5.307	0.060
13	37	62.53	25.53	78.19	46.30	5.256	0.060
14	38.9	70.67	31.77	91.88	50.20	5.219	0.068
15	38.9	74.48	35.58	98.25	51.50	5.189	0.068
16	40.1	77.11	37.01	101.06	53.20	5.176	0.067
17	37.6	72.89	35.29	95.77	50.20	5.187	0.067
18	36.4	67.8	31.43	89.41	47.66	5.214	0.068
19	34.2	59.5	25.27	74.53	43.38	5.257	0.059
20	33.2	51.2	18.03	61.77	39.95	5.308	0.060

### Energy analysis

**Fig. 11:**
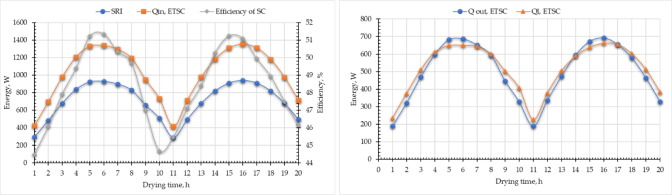
Energy analysis of the evacuated tubes solar collector. Whereas ${\dot{Q}}_{in, \text{ETSC}}$ is the input energy, ${\dot{Q}}_{u,\text{ETSC}}$ is the useful energy, ${\dot{Q}}_{ls, \text{ETSC}}$ is the energy loss, and ${\eta }_{en,\text{ETSC}}$ is the energy efficiency.

Energy analysis of an ETSC refers to the systematic evaluation of its thermal performance by assessing energy inputs, conversions, and losses during the drying process. It examines how efficiently solar radiation is absorbed, converted into heat, and utilized to remove moisture from agricultural or industrial products. This analysis helps optimize SD design, improve energy efficiency, and reduce operational costs while ensuring faster and more uniform drying. By studying factors like heat transfer, airflow, and insulation^74,75^. The energy analysis of the ETSC was shown in Fig. 11, where the ${\dot{Q}}_{in, \text{ETSC}}$, ${\dot{Q}}_{u, \text{ETSC}}$, ${\dot{Q}}_{ls, \text{ETSC}}$, and ${\eta }_{en, \text{ETSC}}$ were calculated hourly based on the SRI and the temperature difference between the input and output of the ETSC. The total ${\dot{Q}}_{in,\text{ ETSC}}$ ranged between 424.8 and 1332 W for the first day and ranged between 416.2 and 1311.8 W for the second day. Figure 11 shows the ${\dot{Q}}_{u, \text{SAC}}$ generated by the ETSC where it oscillates depending on the SRI. Where the ${\dot{Q}}_{u, \text{SAC}}$ was between 189 and 682.5 W (Fig. 11). Higher ${\dot{Q}}_{u, \text{ETSC}}$ values because air exhaust fans run continuously. The ${\eta }_{en, \text{ETSC}}$ of the developed ETSC was calculated based on Eq. (11) then the obtained data was presented in Fig. 11. As shown in Eq. (11), the ${\eta }_{en, \text{ETSC}}$ is directly proportional to the ${\dot{Q}}_{u, \text{ETSC}}$, where it was increased from 44.5% until noon (1 p.m.) it reached 51.2% and then decreased to 44.7% at 5 p.m., according to the oscillation of the SRI. The observed ${\eta }_{en, \text{ETSC}}$ during the current study was compared to previous studies and presented in Table 3.

**Table 3:** Comparison between the obtained ${\eta }_{en, \text{ETSC}}$ with previous studies.

References	Type		${\eta }_{en, \text{ETSC}}$, %
Fudholi et al.^76^	Hybrid solar drying system (HSDS) with rotating rack		40%
Luan et al. ^77^	Multi-pass SAC		52.1%
Rezaei et al. ^78^	SC without phase change material		52.1%
Rezaei et al. ^78^	Bobbin absorber plate without phase change material		36.3%
Rezaei et al. ^78^	SC with phase change material		12.9%
Lingayat et al.^79^	SC with V-corrugated absorption plates		31.50%
Hegde et al. ^80^	Top and bottom flow SC		50.0%
Şevik et al.^38^	Double-pass SC with and without infrared assistance		1.15 and 26.46%
Chowdhury et al.^50^	Tunnel SD		27.45–42.50%
Mathew and Thangavel^81^	Thermal energy storage integrated evacuated tubes		10–30%
Lingayat et al. ^82^	Flat plate SC		45.32%
Current study	The developed ETISD		51.36%

**Fig. 12:**
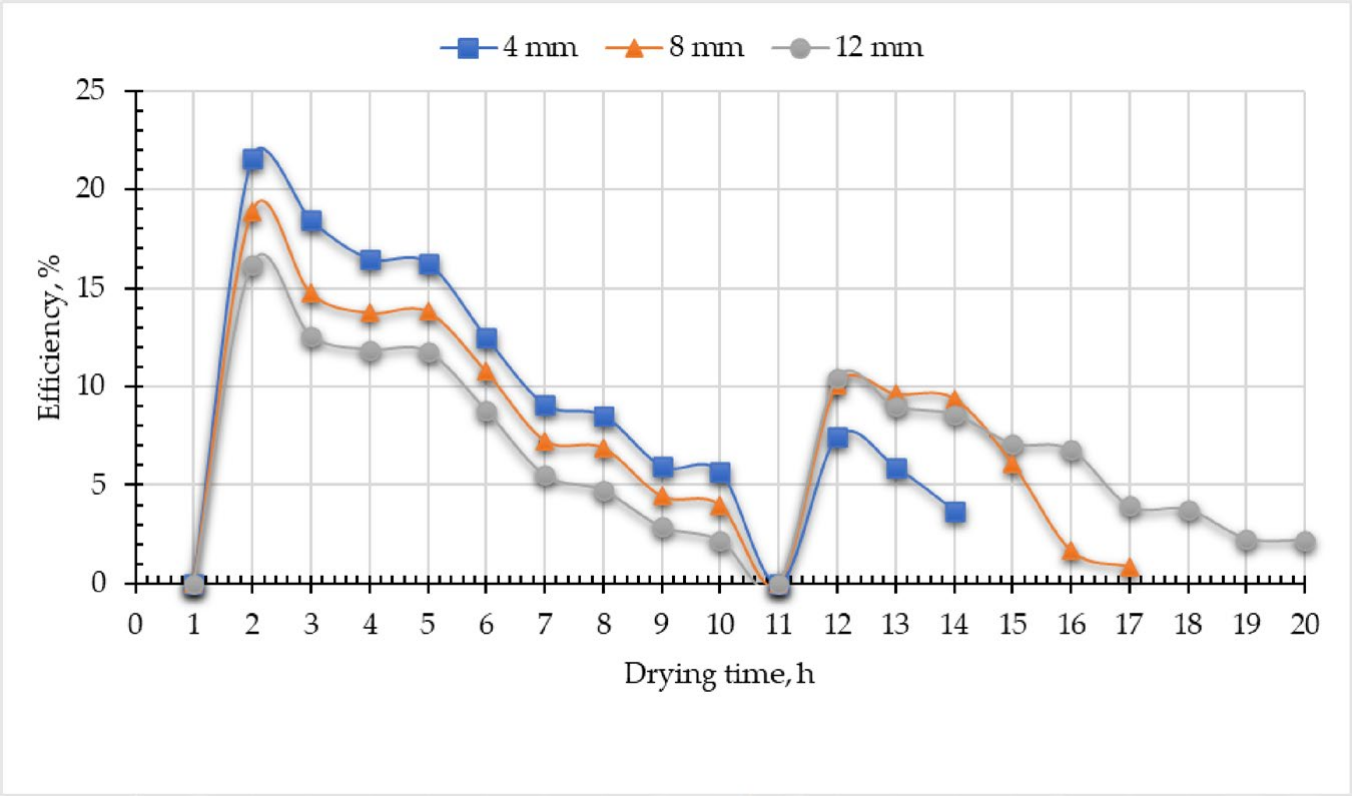
The energy efficiency of the evacuated tubes indirect solar dryer according to the tilapia strips thickness during the drying process at three strip thicknesses of 4, 8, and 12 mm.

Figure 12 shows the ${\eta }_{en, Dryer}$ of the developed ETISD. This analysis evaluates the system’s efficiency in transforming solar energy into usable heat for drying tilapia strips. ${\eta }_{en, Dryer}$ is determined by comparing the energy consumed for moisture extraction across different tilapia samples to the total energy supplied by the ETSC, as computed using Eq. (12). As shown in Fig. 12, the ${\eta }_{en, ETISD}$ was decreased gradually according to the decrease in the moisture content inside the tilapia strips. Where the maximum ${\eta }_{en, ETISD}$ was observed at the beginning of the drying process (8 a.m.) and it ranged between 16.18–21.57%, and the lowest ${\eta }_{en, ETISD}$ was observed at the end of the drying process and it ranged between 2.19 and 6.7%. Additionally, the presented data in the same figure showed that the ${\eta }_{en, ETISD}$ varied according to slice thickness, where the maximum ${\eta }_{en, ETISD}$ was 21.57% at the strip thickness of 4 mm, and the lowest value was 16.18% and it was observed at the strip thickness of 12 mm. this phenomenon due to faster moisture removal. Where reduced thickness shortens the diffusion path for moisture, enabling quicker evaporation. Comparisons between the obtained ${\eta }_{en, \text{ETSC}}$ with previous studies are illustrated in Table 4.

**Table 4:** Comparison between the obtained energy efficiency of the evacuated tubes indirect solar dryer (ETISD) with previous studies.

References	Type	Product	${\eta }_{en, ETISD}$, %
Fudholi et al.^76^	Hybrid SD with rotating rack	Salted silver jewfish	23%
Maia et al.^83^	Baffled SD	Corn	24.9%
Kilanko et al.^84^	Flat plate indirect SD	Scotch bonnet pepper	28.4%
Radhakrishnan et al.^85^	Greenhouse SD	Potato	24%
Radhakrishnan et al.^85^	Greenhouse SD	Eggplant	47%
Radhakrishnan et al.^85^	Greenhouse SD	Apple	49%
Current study	The developed ETISD		51.36%

### Exergy analysis ($\dot{E}x$)

**Fig. 13:**
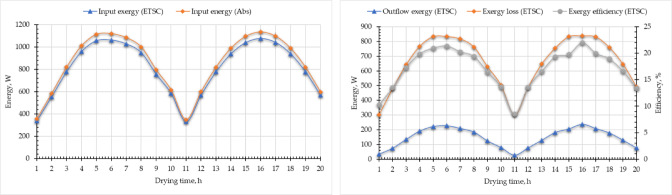
Exergy analysis of the evacuated tubes solar collector. Whereas ${\dot{E}x}_{in, \text{ETSC}}$ is the input exergy, ${\dot{E}x}_{out, \text{ETSC}}$ is the output exergy, ${\dot{E}x}_{ls, \text{ETSC}}$ is the exergy loss, and ${\eta }_{ex, \text{ETSC}}$ is the exergy efficiency.

Exergy analysis is a powerful thermodynamic tool used to evaluate the true efficiency of a SD by assessing not just the quantity but the quality of energy utilization. Unlike conventional energy analysis, which only considers energy inputs and outputs, exergy analysis identifies irreversibilities and losses due to entropy generation, heat transfer inefficiencies, and fluid friction. This method reveals where and how energy degradation occurs by quantifying the usable work potential (exergy) at each stage of the drying process^40,86^. Figure 13 shows the temporal change of ${\dot{E}x}_{in, ETSC}$, ${\dot{E}x}_{out, ETSC}$, ${\dot{E}x}_{ls, ETSC}$, and ${\eta }_{ex,ETSC}$, derived utilizing Eqs. (15–19). Where ${\dot{E}x}_{in, ETSC}$, ${\dot{E}x}_{out, ETSC}$, and ${\dot{E}x}_{ls, ETSC}$ are directly proportional to SRI. As shown in Fig. 13, the ${\dot{E}x}_{in, ETSC}$, ${\dot{E}x}_{out, ETSC}$, ${\dot{E}x}_{ls, ETSC}$, and ${\eta }_{ex,ETSC}$ had the same trend of the SRI, where they increased gradually to reach the peak value at noon and decreased gradually to reach the lowest values at the afternoon. The ${\dot{E}x}_{in, ETSC}$ was estimated based on SRI and it ranged between 330.11 and 805.1 W. While the ${\dot{E}x}_{out, ETSC}$ was calculated according to the temperature difference between inlet and outlet air from the ETSC, and it ranged between 28.09 and 146.4 W.

On the other hand, the ${\dot{E}x}_{ls, ETSC}$ was greater at midday due to the trend of SRI during that time (Fig. 13). Where the average ${\dot{E}x}_{ls, ETSC}$ was 658.7 W, and it ranged between 302.08 and 838.59 W. furthermore, according to Eq. (23), the ${\eta }_{ex, ETSC}$ was calculated presented in Fig. 13. Where the ${\eta }_{ex, ETSC}$ was ranged between 8.51 and 21.99%, and the average ${\eta }_{ex, ETSC}$ was 17.1%. Comparison between the obtained ${\eta }_{ex, ETSC}$ with previous studies was shown in Table 5.

**Table 5:** Comparison between the obtained exergy efficiency of the evacuated tubes indirect solar dryer (ETISD) (${\eta }_{ex, ETSC}$) with previous studies.

References	Type	${\eta }_{ex, SAC}$
Mugi and Chandramohan^87^	Forced convection indirect SD	2.44%
	Natural convection indirect SD	2.03%
Tiwari and Tiwari^56^	Hybrid mixed mode greenhouse SD	19.11–28.96%
Abdelkader et al.^57^	Carbon nanotubes-based SD	8.1–11.9%
Gari et al.^72^	Double air pass solar tunnel SD	9.41%
Lingayat et al.^82^	Using indirect type natural convection SD	7.4–45.23%
Current study	The developed ETISD	21%

**Fig. 14:**
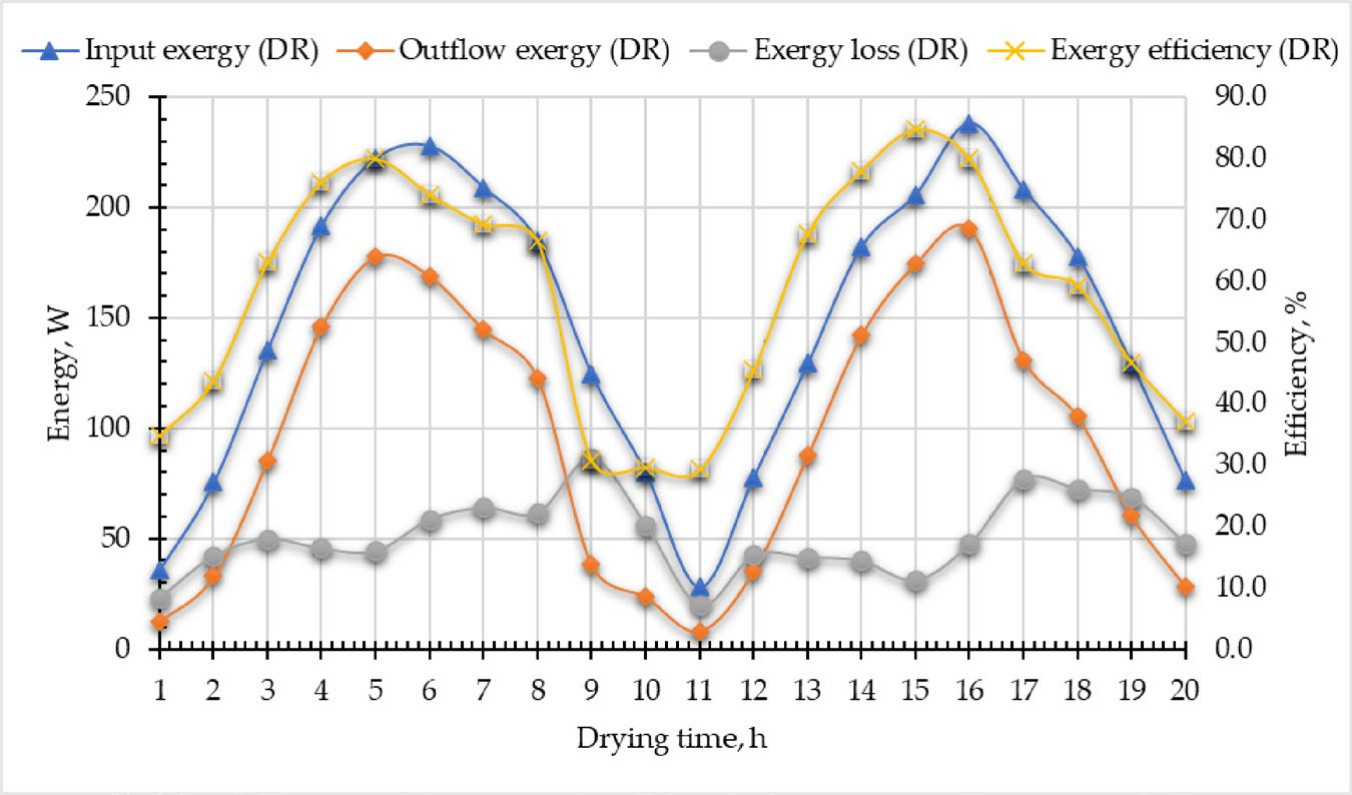
Exergy analysis of the drying room. Whereas ${\dot{E}x}_{in, DR}$ is the input exergy, ${\dot{E}x}_{out,DR}$ is the output exergy, ${\dot{E}x}_{ls, DR}$ is the exergy loss, and ${\eta }_{ex, DR}$ is the exergy efficiency.

The Eqs. (20–23) were employed to compute the ${\dot{E}x}_{in, DR}$, ${\dot{E}x}_{out,DR}$ and ${\dot{E}x}_{ls, DR}$, and the results were plotted according to drying time in Fig. 14. In the current study the manifold of the evacuated tubes lies inside the drying room, where the outlet hot air from the ETSC is the inlet air to the SR, and has the same temperatures. As explained above, the ${\dot{E}x}_{in, DR}$, ${\dot{E}x}_{out,DR}$ and ${\dot{E}x}_{ls, DR}$ were affected directly by the SRI, and maximum values for each parameter were recorded at noon and the lowest values were recorded at the afternoon. According to the ${\dot{E}x}_{in, DR}$, it was dependent on the outlet air temperature from the ETSC, and it ranged between 28.18 and 238.27 W. Additionally, both ${\dot{E}x}_{out,DR}$ and ${\dot{E}x}_{ls, DR}$ were in the range of 8.24–177.8 W and 19.94–86.45 W, respectively. Furthermore, The ${\eta }_{ex, DR}$ ranged between 29.23% and 84.76%. The ${\eta }_{ex, DR}$ exhibited a progressive increase over time, primarily due to the reduced temperature drop of the drying air as the process advanced. This trend occurs because moisture removal from the product diminishes toward the final drying stages, leading to less evaporative cooling and, consequently, a smaller decline in air temperature. As a result, the system retains more usable thermal energy (exergy) at later stages, improving efficiency. Furthermore, the plotted data revealed that the ${\eta }_{ex, DR}$ on the second day consistently surpassed values recorded at the same time intervals on the first day. This difference suggests cumulative thermal gains or improved system stabilization, such as reduced heat losses or enhanced airflow dynamics, during prolonged operation. Comparison between the obtained ${\eta }_{ex, DR}$ with previous studies was shown in Table 6.

**Table 6:** Comparison between the obtained exergy efficiency of the drying room (DR) (${\eta }_{ex, DR}$) with previous studies.

References		Type	${\eta }_{ex, DR}$
Shringi et al.^51^		SD using phase change material as energy storage	67.06–88.24%
Panwar^52^		Natural convection SD	55.35–79.35%
Kesavan et al.^54^		Triple-pass SD	2.8–87.02%
Chowdhury et al.^50^		Tunnel SD	41.42%
Mathew and Thangavel^81^		A novel thermal energy storage integrated evacuated	10–30%
Karthikeyan and Murugavelh^55^		Mixed mode forced convection tunnel SD	23.25–73.31%
Current study		The developed ETISD	29.2 and 84.8%

### Sustainable indicators

**Fig. 15:**
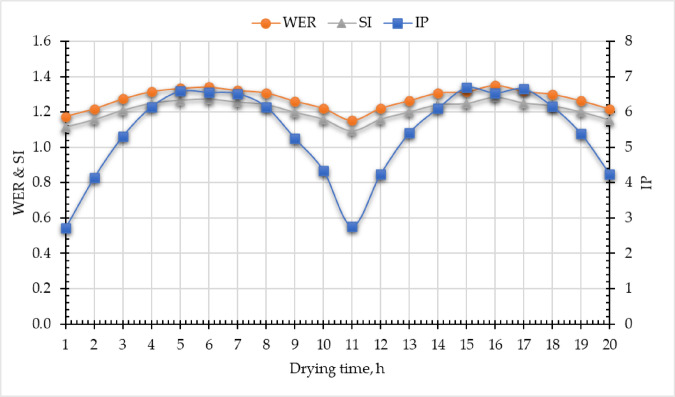
Sustainability indicators of the developed evacuated tubes indirect solar dryer.

The exergy-based sustainability indicators—Improvement Potential (IP), Waste Exergy Ratio (WER), and Sustainability Index (SI)—serve as critical tools for evaluating system performance and optimizing the design of the DR. By analyzing these metrics, engineers can identify inefficiencies, reduce energy waste, and enhance the sustainability of the drying process, thereby promoting more eco-friendly and resource-efficient operations. IP is a key metric within exergy sustainability analysis, quantifying the scope for enhancing the ${\eta }_{ex, DR}$ while minimizing WER. It evaluates the gap between current performance and ideal thermodynamic conditions, guiding targeted efforts to reduce ${\dot{E}x}_{ls, DR}$. By highlighting inefficiencies, IP supports sustainable development, resource optimization, and the transition toward cleaner, high-efficiency energy systems. Mathematically, IP is derived from exergy dissipation (Eq. 24). In this study, observed IP values ranged from 2.71 to 6.69 W (Fig. 15), indicating relatively low exergy losses in the current DR configurational testament to its optimized design. The observed IP values align with the previous studies 12.75 W^47^, and 17 W^88^. While Eqs. (25 and 26) were used to compute both WER and SI. Where the WER ranged between 1.15 to 1.36, this value was slightly higher than previous studies, 0.41 and 0.445^47^, and 0.38 and 0.55^88^. Moreover, the SI value ranged from 1.09 to 1.28. These results lie in the range 1.26 to 1.71^49^, 1.12 to 2.57^86^, and 1.30^75^.

## Conclusion

In the present study, an ETISD was employed to dry tilapia fish strips at three different thicknesses: 4 mm, 8 mm, and 12 mm. To comprehensively evaluate the system’s performance, CFD, energy and exergy analyses, and sustainability indicators were utilized. CFD simulations were conducted to analyze airflow pathlines, temperature uniformity, and velocity vectors within the DR at five different air velocities (0.02, 0.03, 0.04, 0.05, and 0.06 m/s). These simulations aimed to identify the optimal air flow rate that provides an adequate drying temperature for tilapia strips. The initial MC of the tilapia strips was approximately 74.83% (w.b.), which decreased by 18.84%, 18.80%, and 19.45% after 15, 17, and 20 h of drying for slice thicknesses of 4 mm, 8 mm, and 12 mm, respectively. CFD results indicated that setting the exhaust fan to an air flow rate of 0.03 m^3^/s produced an optimal drying temperature of approximately 74.82 °C, at solar noon (around 1 p.m.). CFD simulations, conducted over two drying days from 8:00 a.m. to 5:00 p.m., were instrumental in characterizing the internal fluid dynamic behavior of the dryer. Energy analysis showed maximum thermal energy input, and useful energy output values of 1311.8 W and 682.5 W, respectively. The energy efficiencies ranged between 44.5–51.2% for the ETSC and 16.18–21.57% for the ETISD. Exergy efficiencies ranged from 8.51–21.99% for the ETSC and 29.23–84.76% for the ETISD. Furthermore, sustainability indicators including improvement potential (IP), waste exergy ratio (WER), and sustainability index (SI) ranged from 2.71 to 6.69 W, 1.15 to 1.36, and 1.09 to 1.28, respectively, reflecting the system’s efficiency and environmental viability.

## Data Availability

All data are presented within the article.

## Abbreviations

ETISD: Evacuated tube indirect solar dryer
CFD: Computational fluid dynamics
SAC: Solar air collector
DR: Drying room
ETSC: Evacuated tube solar collector
SRI: Solar radiation intensity
IP: Improvement potential
WER: Waste exergy ratio
SI: Sustainability index
SD: Solar dryer
PCM: Phase change material
PV: Photovoltaic
MC: Moisture content

## List of symbols

$$MC$$: Moisture content
$$W$$: Sample weight
${\dot{m}}_{a}$: Mass flow rate
${\dot{E}}_{a}$: Energy flow rate
$${h}_{a}$$: Enthalpy
$${v}_{a}$$: Air velocity
$${z}_{a}$$: Height
$$g$$: Gravity acceleration
$$\dot{W}$$: Work done
$$\dot{Q}$$: Heat transfer
$${\dot{Q}}_{u}$$: Useful energy
$${\dot{Q}}_{in}$$: Input energy
$${\dot{Q}}_{ls}$$: Energy loss
${I}_{s}$: Solar radiation intensity
${A}_{SAC}$: Surface area of the solar collector
${C}_{pa}$: Specific heat of air
$${T}_{c}$$: Air temperature
$${\eta }_{en}$$: Energy efficiency
${m}_{w}$: Quantity of removed water from date sample
$L$: Latent heat of vaporization of water
$${t}_{d}$$: Drying time
$${\eta }_{ex}$$: Exergy efficiency
$$u$$: Internal energy
$$s$$: Entropy
$\alpha$: Absorptivity of glass
$$\dot{E}x$$: Exergy
$\tau$: Transmissivity of glass
$${\mu }_{ch}$$: Chemical energy
$${T}_{0}$$: Atmospheric temperature

## Subscripts

$$i$$: Inlet
$$o$$: Outlet
$\text{SAC}$: Solar air collector
Dryer: The solar dryer
DR: Drying room
